# Expanding applications of allogeneic platelets, platelet lysates, and platelet extracellular vesicles in cell therapy, regenerative medicine, and targeted drug delivery

**DOI:** 10.1186/s12929-023-00972-w

**Published:** 2023-09-14

**Authors:** Thierry Burnouf, Ming-Li Chou, David J. Lundy, Er-Yuan Chuang, Ching-Li Tseng, Hadi Goubran

**Affiliations:** 1 Graduate Institute of Biomedical Materials and Tissue Engineering, College of Biomedical Engineering, Taipei Medical University, 250 Wu-Xing Street, Taipei, 11031 Taiwan; 2 International Ph.D. Program in Biomedical Engineering, College of Biomedical Engineering, Taipei Medical University, Taipei, Taiwan; 3 International Ph.D. Program in Cell Therapy and Regenerative Medicine, College of Medicine, Taipei Medical University, Taipei, Taiwan; 4 Saskatoon Cancer Centre and College of Medicine, University of Saskatchewan, Saskatchewan, Canada; 5 Present Address: Institute of Clinical Medicine, National Yang-Ming Chiao Tung University, Taipei, Taiwan

**Keywords:** Platelet, Allogeneic platelet concentrate, Human platelet lysate, Extracellular vesicles, Regenerative medicine, Cell therapy

## Abstract

Platelets are small anucleated blood cells primarily known for their vital hemostatic role. Allogeneic platelet concentrates (PCs) collected from healthy donors are an essential cellular product transfused by hospitals to control or prevent bleeding in patients affected by thrombocytopenia or platelet dysfunctions. Platelets fulfill additional essential functions in innate and adaptive immunity and inflammation, as well as in wound-healing and tissue-repair mechanisms. Platelets contain mitochondria, lysosomes, dense granules, and alpha-granules, which collectively are a remarkable reservoir of multiple trophic factors, enzymes, and signaling molecules. In addition, platelets are prone to release in the blood circulation a unique set of extracellular vesicles (p-EVs), which carry a rich biomolecular cargo influential in cell–cell communications. The exceptional functional roles played by platelets and p-EVs explain the recent interest in exploring the use of allogeneic PCs as source material to develop new biotherapies that could address needs in cell therapy, regenerative medicine, and targeted drug delivery. Pooled human platelet lysates (HPLs) can be produced from allogeneic PCs that have reached their expiration date and are no longer suitable for transfusion but remain valuable source materials for other applications. These HPLs can substitute for fetal bovine serum as a clinical grade xeno-free supplement of growth media used in the in vitro expansion of human cells for transplantation purposes. The use of expired allogeneic platelet concentrates has opened the way for small-pool or large-pool allogeneic HPLs and HPL-derived p-EVs as biotherapy for ocular surface disorders, wound care and, potentially, neurodegenerative diseases, osteoarthritis, and others. Additionally, allogeneic platelets are now seen as a readily available source of cells and EVs that can be exploited for targeted drug delivery vehicles. This article aims to offer an in-depth update on emerging translational applications of allogeneic platelet biotherapies while also highlighting their advantages and limitations as a clinical modality in regenerative medicine and cell therapies.

## Introduction

For a long time, allogeneic platelet concentrates (PCs) have been used in transfusion medicine to prevent or treat bleeding episodes in patients with low platelet counts or platelet dysfunction [1, 2]. Over the years, autologous platelet-rich plasma (PRP) gained increasing–sometimes empiric–popularity, used alone or in combination with biomaterials, as adjunct treatment for various pathologies that required support for healing soft and hard tissues and cartilage [3–5]. Recently, however, interest has sharply grown in evaluating the potential clinical translation of biomaterials derived from allogeneic PCs, such as human platelet lysates (PLs; HPLs), platelet extracellular vesicles (EVs; p-EVs), and formulated platelets [6–8], for applications in cell therapy [6, 9–12], regenerative medicine [13–15], and targeted drug delivery (TDD) [16] as needed for aging societies.

Several reasons contribute to the growing interest in platelet biomaterials made from allogeneic donations rather than autologous sources. First, certain patients may face challenges in donating 50–100 mL of their own blood due to various health conditions, lack of venous access, the presence of comorbidities such as thrombocytopenia (a low platelet count), platelet dysfunctions, diabetes, peripheral arterial and neuropathic disease [17–19], or the use of antithrombotic medications, which can impact the blood-clotting process and platelet functions [20]. Secondly, the variability in autologous platelet derivatives, affecting growth factor content and immune cell and inflammatory cytokine profile, further complicates their use [21]. Lastly, significant variations in platelet count enrichment depending on the type of medical devices and procedures used for isolation contribute to the complexity of autologous platelet-derived biomaterials [21–23]. Considering these challenges, exploring the benefits and addressing the challenges associated with allogeneic sources to prepare platelet biomaterials as “off-the-shelf” products becomes an appealing approach for further investigation [24]. The successful use of allogeneic PRP has been previously documented in the treatment of chronic skin wounds, diabetic foot ulcers [17, 18, 25–27], and osteoarthritis [28]. Similarly, allogeneic serum has shown therapeutic value for dry eye syndrome and other ocular surface pathologies [26, 29–31] especially in situations where autologous materials was unsuitable or inconvenient. Notably, there is a growing interest in the use of allogeneic PCs obtained from healthy donors who meet stringent donation criteria and are processed by blood establishments following licensed monitoring procedures adhering to good manufacturing practices (GMPs) principles and meeting consistent quality criteria [9, 32]. Moreover, the use of allogeneic PCs can offer a more cost-effective alternative compared to autologous sources, as the latter often requires additional handling and testing that can increase overall cost. In contrast, allogeneic PCs undergo thorough screening and testing procedures in regulated blood establishments [33], ensuring a high level of safety as source material of biomaterials.

These developments have involved academic researchers, clinicians, as well as medical device, biotechnology, and cell and biotherapy industries, highlighting the strong translational potential and clinical expectations. Such translational developments have benefited from the vast scientific, medical, and regulatory experience gained over several decades by the well-established blood-transfusion and plasma-fractionation community in supporting scientifically based and safe production and clinical use of allogeneic blood-derived cell and protein products [1, 34], as highlighted throughout this manuscript.

The last few years have seen a growing recognition that allogeneic PCs prepared by blood establishments can be repositioned as a valuable source for various biotherapies. The primary aim of this article is thus to provide a comprehensive overview of the scientific, clinical, and regulatory rationale for considering allogeneic platelets as a valuable cellular source material in human medicine. Similar to mesenchymal stromal cells (MSCs) and other cell types, allogeneic platelets hold significant potential for novel applications. In this context, we conduct a critical analysis of the recent advancements in clinical applications involving allogeneic platelets, HPLs, and p-EVs) in the fields of cell therapy (as xeno-free supplement of growth media used to expand therapeutic cells), regenerative medicine, and as targeted drug delivery systems (TDDS).

Throughout this review, we not only emphasize the various advantages and constraints associated with these translational developments but also draw valuable lessons from the blood transfusion and plasma fractionation industry, which can contribute to the safe and effective translational development of allogeneic platelet-based therapies. By highlighting the safety measures and quality control protocols applied in the blood and plasma products industry, we aim to provide valuable insights into ensuring the safety and efficacy of allogeneic platelet-derived biotherapies. In addition to examining the current state of the field, we also explore the potential future therapeutic applications of allogeneic platelets, HPLs, and p-EVs. By encompassing a broad range of topics, this article aims to serve as a comprehensive resource for researchers, clinicians, and stakeholders looking at novel application of platelets.

## Platelet structure, functions, and clinical use

### Platelets: essential therapeutic cells for bleeding disorders and beyond

Understanding the structural and functional roles of platelets under physiological and pathological conditions allows us to realize the growing therapeutic value of platelet-derived biomaterials and biotherapies in the fields of cell therapy, regenerative medicine, and TDD. The scientific rationale for these applications derives from the potent functionality of platelet membranes and the richness of their contents.

Platelets are the first blood cells to respond to an injury and play critical roles in blood clotting, wound healing, and tissue repair. They are, after red blood cells (RBCs), the second most abundant cells in blood circulation with a number ranging (150–400) × 10^6^ cells/mL in healthy human individuals [35], and account to close to 5% of total cells in the body [36]. Resting platelets have a lenticular shape with a diameter of 2–4 μm and a thickness of 0.5 μm. Platelets have a relatively short residence time of 7–10 days in the blood circulation under normal physiological conditions and are continually produced by megakaryocytes in the bone marrow for release into the blood as anucleated cells.

Platelets are known most specifically for their vital function in maintaining blood hemostasis. This primarily role of platelets explains why PCs collected from allogeneic blood donors are essential in transfusion medicine to treat bleeding disorders resulting from thrombocytopenia and platelet function defects [37]. As such, platelet concentrates are featured on the World Health Organization (WHO)'s model lists of essential medicines for adults and children [38]. Our understanding of platelet biology, storage methods, and transfusion techniques has significantly improved, leading to the establishment of standardized protocols for PC production from allogeneic donors and evidence-based transfusion therapy [32]. Various international guidelines and studies have been developed to ensure clinicians’ best practices in transfusing PCs to safely and efficiently address bleeding complications and maintain hemostasis in individuals with platelet-related disorders or those undergoing surgical procedures [2, 37, 39]. A similar approach should be followed to guide the production and clinical use of allogeneic platelet biomaterials, including HPLs and p-EVs.

### Biochemical bases of platelets’ functions in hemostasis, blood coagulation, tissue repair, and immunity

**Fig. 1:**
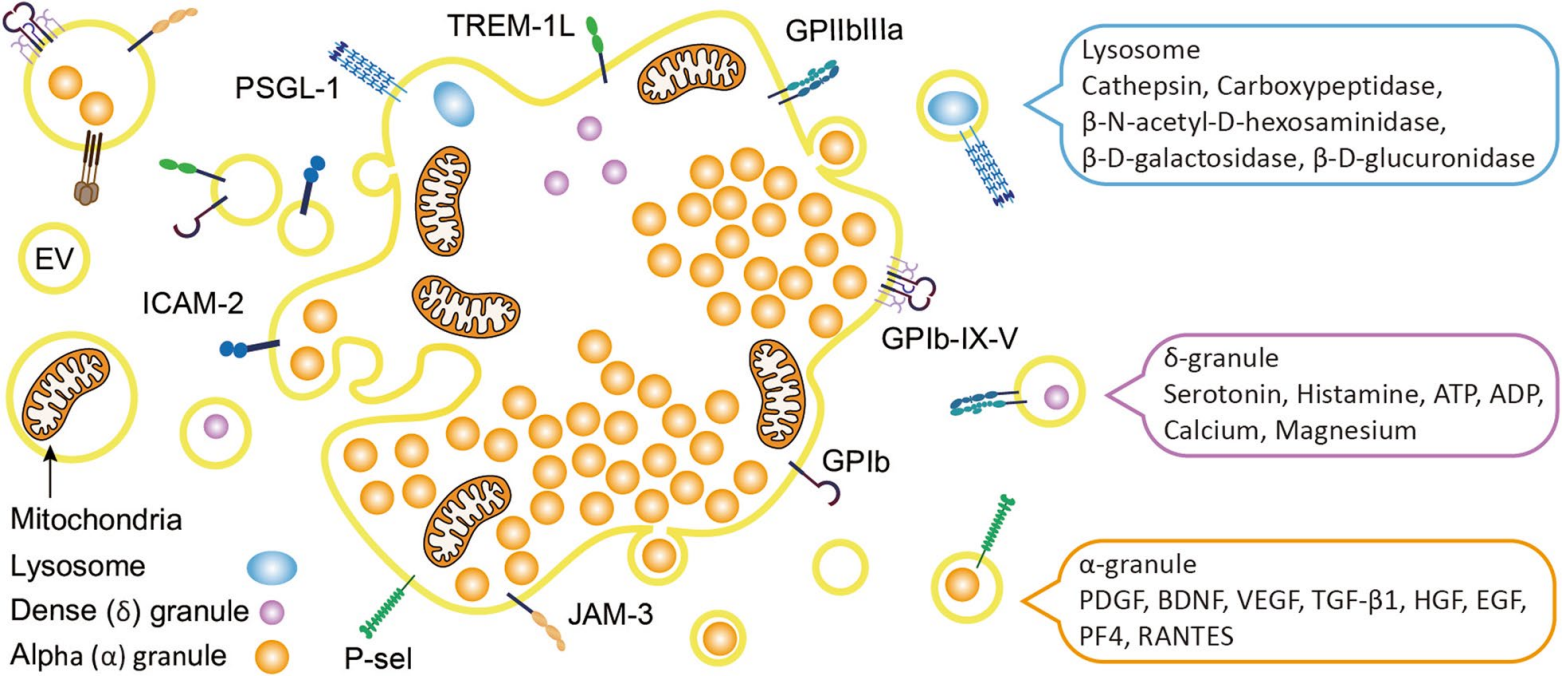
Schematic illustration of the structure, important membrane markers, and cargo of platelets, platelet granules, and platelet extracellular vesicles. *ADP* adenosine diphosphate, *ATP* adenosine triphosphate, *BDNF* brain derived neurotrophic factor, *EV* extracellular vesicles, *GP* glycoprotein, *HGF* hepatocyte growth factor, *ICAM* intercellular adhesion molecule, *JAM-3* junctional adhesion molecule, *PF4* platelet factor 4, *P-sel* P-selectin, *PDGF* platelet-derived growth factor, *PSGL-1* P-selectin glycoprotein ligand-1, *RANTES* regulated upon activation, normal T-cell expressed and secreted, *TGF-B1* transforming growth factor-ß1, *TREM* triggering receptors expressed on myeloid cells, *VEGF* vascular endothelium growth factor

Platelets play a crucial role as the keeper of the integrity of the blood vasculature due to the intimate involvement of their membrane receptors at various stages of the blood-coagulation cascade [40], a series of biochemical reactions that occur in the body in response to injury or damage to blood vessels. It is a complex biochemical process that involves activation of several clotting factors and ultimately results in the conversion of fibrinogen into fibrin, and the formation of a thrombus that helps stop bleeding. The blood coagulation cascade is traditionally divided into two main pathways: an intrinsic pathway, which is activated by contact of blood with a negatively charged surface, such as the surface of a damaged blood vessel, and an extrinsic pathway which is activated by the release of tissue factor (TF) from damaged tissues [40]. Both converge to form thrombin, which converts fibrinogen to fibrin, and leads to a hemostatic platelet fibrin plug [35, 40–44]. A schematic representation of platelets and their biochemical content is provided in Fig. 1.

#### Platelet membranes

Platelets have a highly functional and dynamic external membrane that expresses various glycoproteins, integrins, and antigens [45]. These membrane components are pivotal in orchestrating the complex crosstalk existing between platelets and subendothelial structures exposed by an injured blood vessel wall. Biomolecules expressed on platelet membranes also interact with plasma coagulation factors and activators, and protein components of fibrin clots. Membrane glycoproteins are instrumental in platelet adhesion and platelet activation, and recognize blood clotting factors. GPIIb/IIIa, GPIb-IX-V, GPVI, and P2Y12, which are vital in the hemostatic process preceding the wound-healing phase, are highly expressed by platelet membranes [44]. In particular, GPIIb-IIIa (also known as integrin alphaIIb-beta3 or cluster of differentiation 41 (CD41)/CD61, a specific platelet marker), GPIb-IX-V, and GPVI bind to fibrinogen, von Willebrand factor (vWF), and collagen, respectively. Also, P-Selectin, which is expressed by activated platelets, binds to P-selectin glycoprotein ligand (PSGL)-1 which is present, in particular, on leucocytes. The P2Y12 receptor binds to adenosine diphosphate (ADP) which leads to platelet activation and the release of platelet molecules. The lipid bilayer membrane contains negatively charged phospholipids such as phosphatidyl serine (PS), which triggers coagulation upon platelet activation. The rapid exposure of PS upon platelet activation leads to the binding of the activated coagulation factors Va, VIIa, and Xa, the formation of a prothrombinase complex, and the rapid generation of thrombin that converts fibrinogen into the hemostatic three-dimensional (3D) fibrin scaffold enriched with platelet trophic factors (see below) [40, 42, 43]. This natural fibrin-trophic factor biomaterial sustains tissue repair and remodeling by stimulating “in situ” chemotaxis. The activation process of platelets, which leads to their “degranulation”, is accompanied by a sudden change in structure, a fourfold increase in size, the formation of pseudopods, the translocation of receptors, and the secretion of synergistic trophic factors that are stored in inner granules [35].

It is also now well-accepted that various populations of functional EVs are released during this platelet-activation process and can contribute to coagulation and, beyond that, to inflammation but also tissue repair functions [46, 47]. This dynamic phenomenon occurs because the platelet submembrane cytoskeleton is made of actin filaments and myosin and is supported by microtubules present in the sol–gel zone. Microtubules help move the dense granules and alpha granules in the center of the platelets and facilitate the release of their contents upon activation.

#### Platelet cargo

Platelets contain a rather complex mix of organelles: mitochondria (about five per platelet), lysosomes (one or two/platelet), 150-nm dense granules (ca. five to eight/platelet), and 200–500-nm alpha-granules (ca. 50–80/platelet) and a tubular system that is similar to the endoplasmic reticulum (ER) [45, 48, 49]. The various granules allow the specialized storage of bioactive substances (Fig. 1), which at least for alpha-granules is differentially guided by megakaryocytes [50]. Recent proteomic analyses estimated the number of proteins in platelets to exceed 5000, and platelets were demonstrated to have the capacity to perform some limited translational processes in spite of lacking a nucleus [51]. Mitochondria are vital as a source of energy for platelet metabolism [44, 52]. Lysosomes contain various hydrolases (glucosidase and galactosidase) and proteolytic enzymes (cathepsin D and E, elastase, collagenase, lysosomal-associated membrane protein (LAMP)-2, and CD63 tetraspanin) [45]. Dense granules store adenosine triphosphate (ATP), ADP, Ca^++^, polyphosphate, magnesium, and several bioactive amines like the serotonin and dopamine neurotransmitters which originate from the extracellular environment and contribute to amplifying platelet activation [53, 54]. Alpha-granules are small, membrane-bound organelles with a complex composition of hundreds of proteins encompassing coagulation factors, adhesion molecules, inflammatory molecules, and a range of cytokines, chemokines, and growth factors [44, 55, 56]. These proteins are either synthesized by megakaryocytes or loaded within platelets by endocytosis. These contents are essential for hemostasis and play vital roles in the inflammatory response and initiation of wound healing and tissue repair. Multiple growth factors are present in platelets and include platelet-derived growth factor (PDGF)-AA, -AB, and -BB, vascular endothelium growth factor (VEGF), transforming growth factor (TGF)-β, brain-derived neurotrophic factor (BDNF), epidermal growth factor (EGF), basic fibroblast growth factor (b-FGF), connective tissue growth factor (CTGF), and hepatocyte growth factor (HGF). Other functionally important cytokines and chemokines in alpha-granules include C-X-C motif chemokine ligand 4 (CXCL4 or platelet factor 4 (PF4)), CCL5 (RANTES), interleukin (IL)-1, and tumor necrosis factor (TNF), which are involved in the inflammatory response. Other proteins in alpha-granules include adhesion molecules, such as fibrinogen and vWF, and enzymes, such as metalloproteinases which play roles in breaking down the extracellular matrix (ECM) during tissue repair. Overall, platelet alpha-granules are thus a rich source of biomolecules that play critical roles in platelet functions, hemostasis, and tissue repair. Recent experimental studies using mouse megakaryocyte cultures confirmed the suspicion that different subpopulations of alpha-granules, with dedicated compositions, exist because of differential sorting and packaging by megakaryocytes [57]. The release of the alpha-granule content can occur in a specific manner depending upon the triggering factor for platelet activation [58]. Interestingly, platelets possess an open canalicular system (OCS) that occupies roughly 1% of their volume [44, 59] and allows two-way communication between the inner platelet compartment, especially alpha-granules, and the blood. The OCS is a route for exchanging biomolecules with the platelet environment, and leads to the storage of functional biomolecules, such as fibrinogen, within platelet alpha-granules [60]. The OCS also contributes to storing calcium ions, and other free ions, in platelets [19], which is vital for platelet aggregation. Furthermore, the OCS regulates p-EV release in response to platelet aggregation. Platelets also contain micro (mi)RNAs which can regulate gene expressions in target cells [61, 62].

Overall, platelets play critical roles in modulating pro- and anti-inflammatory processes and tissue-repair mechanisms. They respond quickly to injury, and their actions help protect the body from blood loss and promote the healing process. In the last stage of the coagulation cascade when activated platelets are entrapped within the hemostatic fibrin network, which act as a powerful scaffold for cell migration and growth, and they release a myriad of trophic factors (growth factors, chemokines, cytokines, enzymes, signaling molecules, etc.) into the tissue microenvironment. These factors bind to specific receptors on membranes of target cells and modulate signaling pathways and gene expressions [13]. These events are essential to stimulate inflammation, local MSC proliferation and differentiation, and angiogenesis, and thereby orchestrate overall tissue-repair and tissue-remodeling mechanisms. As such, platelets play a pivotal role as the most potent “healing cells” of the body. These functions are supported by the fact that only one-tenth of the one trillion platelets in the blood circulation of healthy individuals is needed to control bleeding, and that many of the components of the platelet inner compartment are not directly involved in primary hemostasis [56, 63].

Numerous studies have demonstrated the leading contribution of platelets in inflammation, atherogenesis, immune defense, tumor growth and metastasis, or neuroinflammation [44, 64–71], and one of the best recognized functional roles of platelets is to sustain tissue repair and wound healing [63, 68]. It should be kept in mind that upon activation, platelets release their cargo of trophic factors and signaling molecules in a soluble and free form or loaded within EVs which express platelet membrane markers [72–74]. This feature supports the rationale that free platelet trophic factors, obtained as platelet lysates, and p-EVs have therapeutic value in cell therapy and regenerative medicine and, as described below, as drug carriers. Table 1 provides an overview of the multifaceted functional roles of platelets in tissue repair.

**Table 1:** Multifaceted roles of platelets in supporting tissue repair

Functional event	Key factors involved	References
Hemostasis/clot formation	Platelets, through their interaction with coagulation factors, are an essential component of a cascade of biochemical reactions leading to the formation of fibrin clots, which help prevent excessive bleeding from injured blood vessels. This platelet–fibrin clot with a tri-dimensional structure acts not only as a temporary hemostatic barrier, but also as a functional scaffold for tissue repair and tissue remodeling	[14, 357, 358]
Growth factor release	Platelets store in their alpha-granules a diverse range of growth factors, such as platelet-derived growth factor (PDGF), transforming growth factor-beta (TGF-β), vascular endothelium growth factor (VEGF), and insulin-like growth factor (IGF), which can be selectively released upon platelet activation in a free form or packaged into extracellular vesicles. These growth factors promote cell proliferation, angiogenesis, and tissue regeneration	[8, 14, 55, 56, 63, 359]
Stimulation of angiogenesis	Platelet-released factors, including VEGF, PDGF, hepatic growth factor (HFG), and basic fibroblast growth factor (bFGF), support the formation of new blood vessels. Angiogenesis helps deliver oxygen, nutrients, and other biological entities, to sites of injury, facilitating tissue repair	[360, 361]
Remodeling of the extracellular matrix (ECM)	Platelets release enzymes and molecules involved in ECM degradation and remodeling, such as matrix metalloproteinase (MMP)-2 and MMP-9. MMP-2 and MMP-9 break down proteins like collagen and facilitate tissue remodeling during wound healing. Platelets also release fibronectin and vitronectin that are instrumental glycoproteins involved in cell adhesion, migration, and wound healing. Fibronectin binds to the ECM and promotes the attachment and migration of cells during tissue repair	[63, 362]
Modulation of immune responses	Platelets interact with immune cells and release immune-modulating molecules, including chemokines and cytokines, which contribute to regulating inflammation and recruiting immune cells to sites of injury	[63, 362]
Recruitment and differentiation of cells	Platelet-derived trophic factors can attract and stimulate the differentiation of various cells involved in tissue repair, such as mesenchymal stem cells and fibroblasts, promoting their migration to and proliferation at sites of injury	[63, 362]
Antibacterial defense	Platelets possess antimicrobial peptides and enzymes that aid in combating bacteria multiplication at sites of tissue damage, reducing the risk of secondary infections	[362–365]

#### Interactions of intact platelets with other cells

While attention in regenerative therapy is currently focused on the use of HPLs or naïve p-EVs, as a source of various trophic factors, known interactions of intact platelets with other cells can justify exploration of new clinical indications. Indeed, the functions of both intact and activated platelets are intimately linked to their ability to interact with other cell types and biomolecules in the body, which support the use of loaded platelet- or p-EV-based therapies as DDSs [8, 16, 75]. For instance, as mentioned above, platelets interact with endothelial cells to form a physical barrier that helps maintain vascular integrity. Activation of the endothelium triggers platelet adhesion and aggregation, which is crucial for hemostasis and wound healing by promoting tissue repair and preventing excessive bleeding. Activated platelets can enhance leukocyte recruitment to sites of inflammation and infection, influencing the immune response, playing an instrumental role in the development of some diseases like atherosclerosis [76, 77], and modulating both innate and adaptive immune responses [78]. Platelets and neutrophils are increasingly recognized as playing synergistic roles, involving the stimulation of neutrophil extracellular traps (NETs), in thromboinflammation and pathogenesis of neuroinflammatory diseases [71]. Additionally, platelets interact with various extracellular matrix components, such as collagen and vWF, which facilitate platelet adhesion and activation during injury or vessel damage and promote tissue regeneration and repair [63]. Platelets play a central role in the coagulation cascade by interacting with coagulation factors, such as fibrinogen and thrombin, leading to clot formation and hemostasis. Thus, therapies using platelets or p-EVs as DDSs can be engineered in a tailor-made fashion to target these interactions and further enhance clot formation or, on the contrary, prevent thrombosis [79]. p-EVs, which carry a diverse cargo of bioactive molecules, can be released by platelets during interactions with other body cells, including cancer cells [80]. These p-EVs can interact with target cells and modulate cellular processes.

The understanding and control of platelet interactions with different cell types and biomolecules may thus help tailor therapies for specific clinical applications in regenerative medicine and cellular/subcellular therapies to promote tissue repair, regulate immune responses, enhance angiogenesis, modulate coagulation, and treat pathologies.

## Allogeneic PC collection and properties

### Collection procedures

Platelet derivatives, including PRP and “platelet gels” used for regenerative medicine, are still typically obtained from autologous patient-specific blood fractions [81, 82]. However, there is now an increasing focus on considering allogeneic (donor-derived whole blood or apheresis) PCs prepared by blood establishments and hospital blood banks as starting materials to make platelet-derived biomaterials [17–19, 24–27, 83–86]. Preparation procedures of PCs from allogeneic blood donors by blood establishments (a term equivalent to blood centers) using whole-blood donations or plateletpheresis procedures were described in previous publications [9, 10, 87–89] and are summarized below.

#### Whole blood collection

Allogeneic PCs can be obtained from whole blood donations, where a donor donates by venipuncture a unit of whole blood (typically 200–450 mL depending upon the jurisdiction) that contains RBCs, plasma, and platelets. Whole blood-derived PCs, known as “random-donor” platelets, are obtained as byproducts of RBC preparations. These platelets can be processed by the PRP or the buffy coat (BC) separation procedures [9]. To collect whole blood-derived platelets, 450 mL of whole blood, containing RBCs, white blood cells (WBCs), platelets, and plasma is collected in a polyvinyl chloride bag containing 63 mL of an anticoagulant solution typically composed of citrate, glucose, and adenine. It is important to complete the donation procedure in less than 12–15 min to minimize the risk of activating blood coagulation and platelets. The donated blood can be stored at 22 ± 2 °C for up to 24 h. Indeed, if PCs are being prepared, the blood should not be cooled down to 2–6 °C, as this can result in the loss of platelet viability and the formation of platelet and leukocyte microaggregates. The first centrifugation step of whole blood plays a crucial role in determining PC characteristics. It is important to apply precise conditions of g-forces, acceleration, time, and deceleration to ensure the separation of different blood components and obtain PCs with specific and consistent compositions [90].

In the PRP method, the whole-blood donation undergoes light spinning centrifugation (typically 1000 × *g*) for around 10 min at 21–22 °C, using validated acceleration and deceleration curves. Afterward, the PC is left at room temperature for about 1 h and then resuspended in 50–70 mL of plasma [90]. The PRP method is the predominant approach for producing platelets from whole blood in the United States.

The BC procedure involves subjecting anticoagulated whole blood to vigorous centrifugation at approximately 3000 × *g* for 5 min. Thirty milliliters of plasma is returned to the BC layer and gently mixed before undergoing another round of centrifugation. This time, light spinning centrifugation at approximately 1000 × *g* for 6 min at 21–22 °C is applied, employing validated acceleration and deceleration curves [90]. The resulting supernatant is subsequently placed in a designated storage bag and stored at 22 ± 2 °C. In more-recent techniques, a platelet additive solution (PAS), containing sodium/potassium chloride, citrate, phosphate, and mannitol, can be employed to replace a portion of the plasma. The BC method is particularly favored in Europe due to its ability to effectively deplete leukocytes through BC removal, thereby reducing the incidence of febrile transfusion reactions and facilitating efficient leukodepletion when combined with leukocyte filtration. To obtain an adult therapeutic dose equivalent to that of a single apheresis platelet procedure, it is necessary to pool the platelets collected from four to six whole blood donations.

#### Plateletpheresis collection

Apheresis PCs can be prepared using automated cell separators and a closed collection and processing system. These dedicated extracorporeal procedures employ intermittent or continuous centrifugation to separate and collect platelets. Multiple cell separators with varying collection principles are available for platelet collection [89]. At the point of withdrawal from the donor, whole blood is anticoagulated in a controlled manner with a blood/anticoagulant ratio of 9:1–11:1: Typically, RBCs are returned to the donor, and, potentially, the plasma can be recovered in the separate bag (“concurrent plasma”) and used for clinical applications or fractionation. The volume of such PCs is typically 200–300 mL per donation, with an enrichment in platelets of about 6–eightfold compared to blood. Apheresis PCs are obtained from a single donor and are not pooled for transfusion purposes. During processing, the PC may or may not be subjected to a leukoreduction step, usually by dedicated filtration, to reduce the content of WBCs, and may be formulated in 100% plasma or in a combination of plasma and PAS. Plateletpheresis procedures yield a higher concentration of platelets compared to whole-blood donations. Indeed, a larger number of platelets can be collected in a single plateletpheresis session. The plateletpheresis procedure takes longer donation times and may require specific vein accessibility for the apheresis machine. Plateletpheresis frequency varies based on individual health and regulatory guidelines but is typically up to 24 per year in most jurisdictions [91]. Regardless of the procedure used, production of PC should comply with GMPs to meet established quality, safety and consistency criteria for transfusion use [32].

#### Variables in PC collection

Several variables in PC production methods can influence the platelet, leukocyte, and protein contents. For instance, apheresis PCs usually have significantly lower WBC contamination than standard BC-derived or PRP-derived PCs [92]. Procedures for preparing PCs may include, as mentioned above, a leukoreduction step using different types of leukoreduction filters [93, 94]. When leukocytes are present in PCs, the resulting lysates may contain higher amounts of cytokines. In addition, until recently, PCs were suspended in 100% plasma. However, residual plasma may mediate transfusion-related adverse reactions in recipients when used intravenously. Therefore, PAS may be used to substitute for part, usually 2/3, of the plasma volume [95, 96], decreasing the protein content and the presence of plasma nutrients. Treatments of PC by gamma-irradiation, to inactivate leucocytes and prevent a risk of graft-versus-host disease upon transfusion, or by photochemical treatments, to inactivate pathogens, may affect the platelet proteome or stimulate the release of cytokines [88, 97, 98]. All these variables in the preparation of allogeneic PC may potentially impact their performance in cell therapy or regenerative medicine applications and should be thoroughly recorded and monitored to draw scientifically-based conclusions in the use of platelet-derived biomaterials.

#### Pathogen reduction treatments of PCs

The implementation of pathogen reduction treatment is a determining factor in the preparation of PCs as it may significantly impact the virus safety of pooled platelet-derived materials [99, 100]. Indeed, in some jurisdictions, individual PC donations intended for transfusion can be subjected to a licensed pathogen-reduction step aimed at inactivating bacteria, viruses, and/or parasites. Pathogen reduction is achieved by adding to the PC donation a small molecule, like psoralen [101] or riboflavin [102], that can penetrate cells and nuclei, bind to nucleic acids and, upon UV light activation, induce alterations making the viruses noninfectious [103–106]. Such photochemical treatments are possible because platelets are anucleated. Extensive validation studies conducted by the developers of pathogen-reduction treatments have generally demonstrated their effectiveness in inactivating a wide range of pathogens [107, 108] and reducing the risk of transmission of viruses (e.g., HIV, hepatitis B and C viruses), bacteria, and parasites. However, some limitations in the extent of inactivation of enveloped and non-enveloped blood-borne viruses resistant to UV-based treatments were also reported [109, 110], suggesting that certain viruses may escape complete inactivation when platelet donations contain infectious titers above the inactivation threshold provided by the technique. Such potential residual viral infectivity, which is even more relevant when the PC are not subjected to a pathogen-reduction treatment, raises specific concerns for the viral safety of allogeneic platelet-derived HPLs or p-EVs when they are made from PC pools as more recipients are at risk of a blood-borne infection. Furthermore, these pathogen-reduction steps might not eliminate the risk of transmission of emerging or unknown viruses, as some viruses may exhibit varying levels of susceptibility to the employed technologies. Therefore, countermeasures to enhance the virus safety margins include limitation of the pool size [100], testing of mini-pool and manufacturing platelet pool for relevant virus markers, or additional dedicated virus-reduction treatments [6, 99], as described below.

**Fig. 2:**
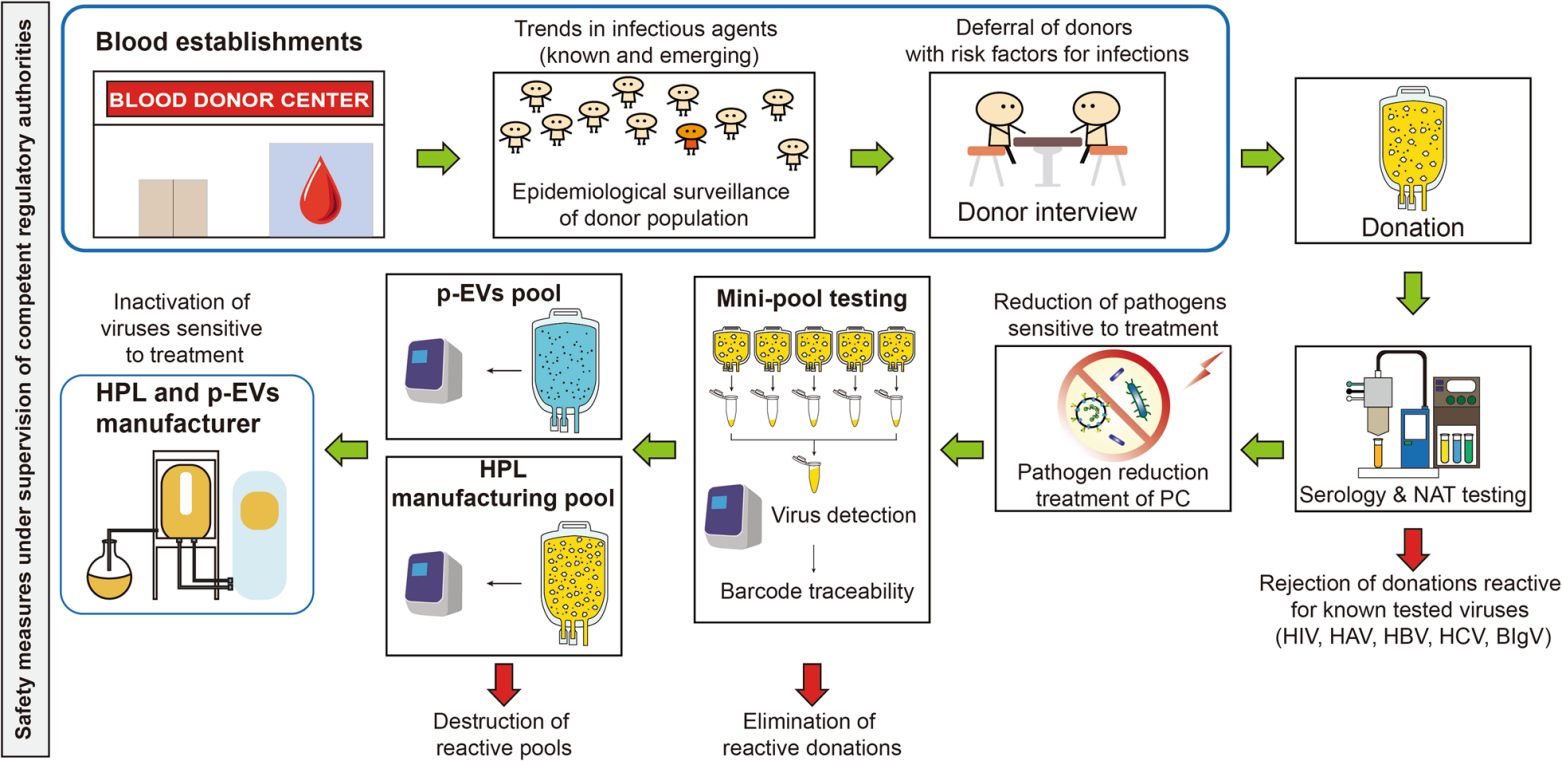
Overview of the various virus safety measures in place in the blood transfusion and industrial plasma fractionation industry, and supervised by regulatory authorities, as potential models to be implemented for optimal virus safety margin of pooled human platelet lysates and platelet extracellular vesicles for clinical applications. *p-EV* platelet extracellular vesicles, *HPL* human platelet lysate, *NAT* nucleic acid testing, *PC* platelet concentrate

The expertise developed by the blood transfusion industry over the last 4 decades in establishing measures to protect the blood supply against known and emerging viruses, including the development, validation, clinical evaluation, licensing, and implementation of dedicated pathogen reduction treatment [103, 111–113], can be of great value to the development of safe and efficient allogeneic PCs as source materials for biomaterials. The various complementary safety measures that can be considered for the production of pooled platelet-derived materials, based on experience developed by the plasma fractionation industry [34], are summarized in Fig. 2.

Additionally, the safety of PCs and the effectiveness of licensed pathogen-reduction treatments of PCs for transfusion are continuously being assessed in advanced countries through hemovigilance programs overseen by regulatory authorities to ensure timely implementation of appropriate countermeasures, such as donor exclusion criteria and screening, as well as donation testing, to minimize the transmission of infectious agents in allogeneic PCs [113–115].

#### Storage of PC

Platelets undergo physiological and functional changes upon storage which may affect therapeutic benefits to the recipient, or further applications in cell therapy or clinical use. PCs are stored at 20–24 °C for up to 5–7 days, depending upon the jurisdiction and specificities of the collection and test procedures [9, 10, 87–89]. They should be stored under continuous gentle agitation in a bag made of cell-compatible plastic with enhanced oxygen permeability and diffusion of carbon dioxide. The pH should remain above pH 6.4. Temperatures below 20 °C may induce platelet activation and release platelet storage components into the plasma. Proper storage conditions limit the risk of activation and maintain an unaltered hemostatic state for intravenous infusion. Agitation enables good mixing and gas exchange. Folding of the bags and foaming should be avoided. Platelets used for transfusion can typically be stored for up to 5–7 days, depending upon national regulations, after which time they can no longer be used for transfusions and are currently discarded. However, such expired (or outdated) PCs can be used as source material to prepare lysates for propagation of therapeutic human cells [6, 116]. The PC are then frozen at − 20 °C or colder until being processed into HPLs.

Table 2 summarizes the most significant characteristics of allogeneic PCs available from blood establishments using current production procedures. Briefly, all therapeutic PC unit should have a total platelet count exceeding 2 × 10^11^. Limits in the acceptable residual leukocyte content (of < 10^9^ to < 10^6^) depend upon whether the PC is stored in 100% plasma or a combination of plasma (30–40%) and additive solution (60–70%), and whether a leukoreduction step is used.

**Table 2:** Characteristics of the various types of allogeneic platelet concentrates prepared by blood establishments for transfusion purposes

Processing method	Suspension and storage solution		Leuco-reduction step	Cell count/PC unit	
	Plasma (%)	Platelet additive solution (%)		Platelets	Leukocytes
Whole blood (buffy coat or PRP methods)	100			> 2 × 10^11^	< 10^9^
	100		Yes		< 10^6^
	30–40	60–70			0.3 × 10^9^
	30–40	60–70	Yes		< 10^6^
Plateletpheresis	100				< 0.3 × 10^9^
	100		Yes		< 10^6^
	30–40	60–70			< 0.3 × 10^9^
	30–40	60–70	Yes		< 10^6^

*PC* platelet concentrate, *PRP* platelet-rich plasma

#### Regulatory oversight of PC collection

In countries with a mature regulatory system, blood establishments responsible for producing blood components, including platelet fractions, should have a strict quality system in place based on GMP principles and should comply with local regulations [117, 118] to ensure the quality and consistency in meeting specifications. Anticoagulants, any additive solutions, and collection and storage bags should be licensed. Preparation equipment should be validated and premises maintained under clean and hygienic conditions and monitored. Preparation of PCs and pooling should be done using aseptic procedures, and either multiple bag configurations or sterile connecting devices used following a validated procedure for closed-system processing. Although variability linked to individual donors cannot be eliminated, such requirements ensure a relative consistency in the characteristics of the various types of allogeneic PCs, particularly platelet and leukocyte counts, used to prepare PLs for MSC expansion, which should contribute to higher reproducibility.

## Advantages and constraints of allogeneic PCs as source materials for platelet-derived biomaterials

**Fig. 3:**
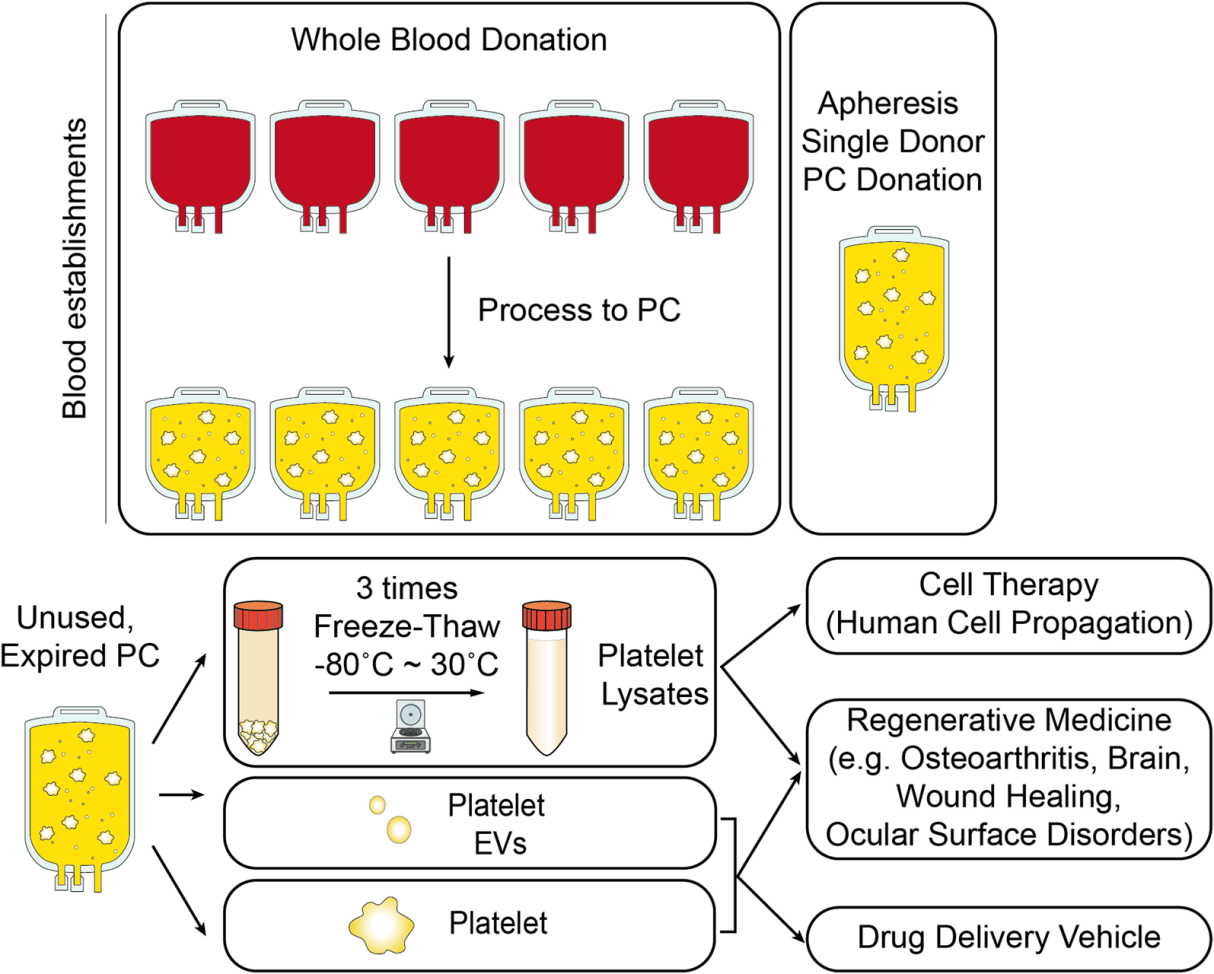
Overview how allogeneic PCs may be used for preparing platelet lysates, extracellular vesicles, or purified platelets for novel applications in cell therapy for human cell propagation, regenerative medicine in various clinical indications, and as drug delivery vehicles. *PC* platelet concentrate, *EVs* extracellular vesicles

Figure 3 provides a typical scheme of the production steps of allogeneic PCs and their processing for preparing different classes of derivatives (HPL, EVs, isolated platelets) for application in cell therapy industry, regenerative medicine, and drug delivery, as discussed in the following section of this article.

Objective reasons justify the interest in using allogeneic PCs to prepare platelet-derived biomaterials for translational cell therapy and regenerative medicine. First, the use of PCs from a donor represents a readily available clinical-grade therapeutic source that is highly valuable when the patients themselves are physically unable or unwilling to donate blood for their own treatment [17–19]. In addition, allogeneic PCs donations are available in substantially larger volumes (ca. 250 mL) than autologous PCs (20–100 mL), which allows for a wider range of clinical benefits, including larger dose sizes and multiple administrations to patients. One valid argument often mentioned supporting autologous platelet donations is the absence of risks of pathogen transmission. However, in a regulated blood-collection setting from volunteer-screened repeat blood donors, as is the case for licensed blood establishments in regulated countries, a single-donor allogeneic PC has a high margin of viral safety [119]. Individual blood donor candidates are screened to assess their suitability for blood donations, and they should have low risk factors for transfusion-transmitted viral infections. When a donor is eligible, dedicated mandatory blood testing of each individual donation is performed to verify non-reactivity (such as the absence of antibody markers or viral nucleic acids) against human immunodeficiency virus (HIV), hepatitis B virus (HBV), HCV, and, in some jurisdictions, other viruses such as West Nile virus or parvovirus B19 [120]. Any reactive donations are discarded, and the blood donor deferred, temporarily or permanently, from subsequent donations depending upon the specificity of the infection identified. Additionally, in wealthy countries with advanced blood-donation systems, PCs are subjected to dedicated pathogen reduction using a licensed photochemical treatment, as mentioned above [99, 100]. The product is then labeled, sealed, and shipped to hospitals for clinical use. Thus, using PCs from blood establishments presents the advantage of guaranteeing safety and consistency in total platelet counts, with 4–sixfold enrichment in platelet counts compared to the baseline level in the blood circulation, and with production methods that can yield minimal residual RBC and white blood cell (WBC) contamination [32]. Controlled operating procedures contribute to minimizing platelet damage, providing an optimal material as a TDD vehicle. Additionally, allogeneic PCs have a well-understood clinical safety and efficacy background for transfusion to trauma and surgical patients, facilitating translation of PCs to emerging clinical indications. Lastly, mounting experimental evidence has shown that 5–7-day outdated allogeneic PCs can be frozen and further processed into derivatives, such as HPLs as a growth medium supplement for human cell propagation [6, 10, 121]. The possibility of processing frozen outdated allogeneic PCs is valuable to facilitate the supply, avoids wastage of precious blood resources, and supports the sustainability of blood establishments as it avoids wastage of this blood component and cost of its destruction [6].

Although allogeneic PCs prepared by a given mode of production provide consistent specifications [32], some variations in preparation procedures, as highlighted above, including use of a PAS in place of plasma [122] or pathogen reduction [103–105], can impact the PC characteristics, and therefore those of biomaterials made from the PCs. For instance, PCs suspended in 100% plasma retain the integral composition and concentration of the native plasma. This implies that HPLs derived from such unprocessed PCs also contain, in addition to platelet components, valuable plasma proteins, such as albumin, immunoglobulins, the full range of coagulation factors, including fibrinogen, and physiologically important anticoagulants, protease inhibitors, and plasma-borne growth factors including most of the IGF-1 present in the blood circulation [123, 124], as well as plasma-borne lipids and EVs [125–128]. This type of HPL, with a total protein content close to 60 mg/ml was found by numerous studies to be optimal for expanding human cells [6]. In contrast, PCs suspended in a mixture of plasma and PAS are partially depleted of plasma protein components proportionally to the extent of the substitution by PAS, but concentrations of platelet-derived components remain unaltered in the corresponding HPLs as the platelet count is unchanged. Experimental data illustrated that the use of PAS can affect the capacity of the resulting HPLs to expand MSCs [129], although their performance in cell propagation still remained superior to that obtained with FBS supplementation [128, 130]. Similarly, the total content of EVs derived from the blood circulation at the time of collection is expected to be higher in PCs suspended in plasma than in a mixture of plasma and PAS, as the collected plasma contains EVs originating from erythrocytes, platelets, megakaryocytes, and leukocytes [131]. Platelets stored in plasma may maintain superior functionality, as plasma provides a physiological environment for preserving platelet viability and activity. On the other hand, platelets stored in PAS may exhibit some functional alterations due to the absence of certain plasma components. One study identified that the type of PAS used can have a significant impact on the level of platelet activation, exposure to CD62P, content of soluble glycoprotein V (sGPV) and the size and number of p-EVs as found by nanoparticle tracking analysis [131]. However, impacts on the storage of platelets in plasma compared to storage in plasma/PAS mixtures or various types of PAS deserve further analysis. From another angle, the use of PAS was recently found to be associated with lower accumulation of complement activation products compared to plasma, which could result in less risk of side effects, at least for clinical transfusions [132], and potentially some therapeutic indications of platelet biomaterials.

Another important factor potentially affecting characteristics of PCs is implementation of pathogen-reduction treatments. Although photochemical inactivation and UV light treatment are considered to have minimal impacts on platelets, it was shown that riboflavin/UVB induced protein upregulation on EVs at day 7 of storage, reflecting the presence of platelet storage lesions potentially predictive of adverse transfusion reactions [133]. In conclusion, the choice of preparation method and pathogen-reduction steps can influence the storage duration and conditions of PCs and their derivatives. Some methods may extend the shelf life of platelets by reducing the risk of bacterial transmission, allowing for longer storage periods, but also possibly impacting platelet viability and functionality and use in cell therapy and regenerative medicine. Documented monitoring of process variables is therefore needed to optimize consistency in functional outcomes of platelet derivatives.

## Use of allogeneic HPL for cell propagation

### Preparation of the platelet lysate or releasate

Allogeneic PCs can be used to prepare “lysates” by breaking down or “releasate” by activating platelets to release their contents of growth factors, cytokines, enzymes, signaling molecules, and other biomolecules valuable for propagating and differentiating therapeutic human cells for transplantation [6, 9, 10] (Fig. 3). This is achieved by combining physical, mechanical, and chemical treatments such as freeze–thaw cycles [134], ultrasound or sonication [135], or solvent/detergent (S/D) [136], respectively, followed by downstream centrifugation and filtration [6, 137]. Fibrinogen seroconversion into fibrin may additionally be performed to prevent growth medium gelation during cell culturing. This can be achieved by adding calcium chloride [6, 10], or preferably a combination of calcium chloride and glass beads as synergistic catalysts of the coagulation [126, 127, 138, 139], thereby avoiding the need for the addition of an anticoagulant such as heparin to avoid gelation of the cell culture medium [10, 121].

### Virus inactivation or removal during HPL processing

Recent articles [99, 100], inspired by vast knowledge on complementary measures needed to guarantee the viral safety of fractionated pooled plasma-derived medicinal products [34, 140, 141], stressed the fact that HPLs, when made from pooled PCs, inherently have statistically higher risks of contamination by blood-borne viruses. It was demonstrated that lysates made from PCs pathogen-reduced by psoralen/ultraviolet (UV) A [142–145], riboflavin/UV treatment [146] or short-wave UV light treatment [147] can maintain their functionality to support the propagation of therapeutic MSC in vitro. This is vital considering that pooling of over 50 donations [6, 12, 148], which increases the risks of contamination by blood-borne viruses [99, 100, 149], has been considered a suitable pool size to provide consistency in HPL quality and improved productivity.

As is the case with the manufacture of pooled plasma protein products, the manufacture of large-pool HPLs requires the application of GMPs and a combination of appropriate procedures to minimize risks of viral transmission to recipients, including platelet donor screening, donation and mini-pool and manufacturing HPL pool virus testing and virus-reduction treatment(s) (Fig. 2). As the efficiency of pathogen-reduction treatments of PC donations has some limits in the range of viruses inactivated, as mentioned above [109, 150, 151], virus-reduction treatments of pooled HPLs must be considered when feasible. Measures implemented should be based on scientific evidence and integrate risk assessments that consider the HPL pool size [99, 100].

Here again, the experience gained with pooled human plasma products is of interest as authorities regulating industrial plasma products recommend manufacturers to implement two robust validated orthogonal (i.e. different and complementary) virus-reduction treatments [152, 153]. The plasma fractionation industry has thus developed several robust virus-reduction procedures applicable to complex or purified plasma protein products. Historically, heat treatment in the liquid state at 60 °C for 10 h, known as “pasteurization”, was applied to human serum albumin, a relatively heat-stable protein, stabilized by low doses of caprylate or acetyltryptophanate to inactivate HBV and other blood-borne enveloped and non-enveloped viruses [154]. Various formulations of stabilizers combining high doses of sugars, polyols, and/or amino acids were also developed to pasteurize coagulation factors, protease inhibitors, and immunoglobulins [154]. In most cases, pasteurization requires a subsequent step of tangential flow filtration to remove the stabilizers [34, 140, 155]. Dry-heat virus inactivation treatment refers to heating a lyophilized protein preparation at temperatures ranging from 60 to 100 °C for durations varying between 0.5 and 96 h depending upon the proteins and the viruses targeted [34, 140, 156]. Low-pH (at ca. pH 4–4.5) treatment in the presence of small quantities of pepsin was demonstrated by the plasma fractionation industry to be a potent virus-reduction treatment of lipid-enveloped viruses in intravenous immunoglobulin preparations [34, 140, 157]. Chemical S/D treatment using combinations of a solvent (tri-n-butyl phosphate, TnBP) and various non-ionic detergents (e.g. Tween-80, Triton X-100, and Triton X-45) at temperatures ranging–20–37 °C for 1–6 h is extremely efficient at inactivating lipid-enveloped viruses and is currently applied to whole plasma [158, 159] and to a large range of PDMPs [160]. The S/D agents can be removed by chromatography and/or oil extraction [140, 158]. Finally, the plasma fractionation industry has largely adopted a dedicated virus-removal step, typically known as “nanofiltration” that consists of filtering plasma protein solutions through specifically designed virus filters with pore sizes of 15, 20, 35 nm, or equivalent [161, 162], to remove enveloped and non-enveloped viruses based on size-exclusion [163]. Among those virus-reduction treatments of plasma products, only solvent/detergent (S/D) can be directly applied to complex protein solutions like HPLs [126, 130, 136, 164] as other methods would most likely denature or precipitate most of HPL components. Other virus-reduction treatments developed for pooled HPLs include gamma-irradiation [146, 164, 165] and electron (e)-beam irradiation [166]. Virus removal by sequential 35–20-nm nanofiltration of 10% HPL-supplemented growth media has been proven to be feasible [128, 167], providing a procedure that also avoids the risks of contaminating the cells by adventitious viruses during media preparation. Combining psoralen/UVA treatment of PC donations and S/D treatment of pooled HPLs was shown to be technically feasible [130], as is nanofiltration of growth media supplemented with HPLs made from psoralen/UVA-treated PCs [128]. These methods can effectively inactivate or remove a broad range of enveloped and non-enveloped viruses, including HIV, HBV, HCV, West Nile virus, hepatitis A virus (HAV), and parvovirus B19. Therefore, technical solutions exist to ensure the needed margin of safety of large-pool HPLs used for human cell propagation, thus complying with existing regulations of blood-derived products [99, 100]. Additionally, the starting HPL donations, assembled as an experimental mini-pool, and HPL manufacturing pool can be tested to confirm that virus contamination is not detectable, as done in the plasma-fractionation industry [34]. Mini-pool testing makes it possible to eliminate reactive donations prior to assembling the whole donations into a large-scale manufacturing pool, therefore serving as a precautionary measure to limit the risks of destroying a manufacturing pool ultimately found to be contaminated by a virus (Fig. 2).

### Allogeneic HPL-based propagation of human cells for cell therapy

HPL can serve as a substitute for FBS as a supplement in growth media for propagating various therapeutic cells, with most of the experimental data obtained thus far focused on MSC expansion, but now expanding to differentiated cells and immune cells [121], as explained below.

HPLs made from clinical-grade allogeneic PCs, including those that are “outdated” (i.e., after 5–7 days of storage at 20–24 °C after collection), are a proven efficient growth medium supplement to successfully substitute for FBS for in vitro xeno-free MSC expansion [6, 10–12]. MSCs can be isolated from bone marrow and adipose tissues and expanded successfully in HPLs, exhibiting proliferation, immunophenotype, and differentiation properties that meet the criteria of the International Society of Cell and Gene Therapy. MSCs expanded in media supplemented with HPLs grow faster than in FBS (meaning a shorter doubling time), maintain clonogenicity and typical MSC immunophenotypes, and exhibit an unaltered differentiation capacity into the three referenced lineages (chondrocytes, osteocytes, and adipocytes) as well as immunosuppressive functionality [137, 149, 165, 168]. Better performance using HPL than FBS may possibly be due to the human origin of the HPL and/or the higher concentration of platelets in PC than in bovine blood, resulting in a higher concentration of growth factors. It was observed in some studies that supplementation with HPLs versus FBS favors MSC differentiation into chondrocytes [126, 128, 130, 169]. HPLs are also a suitable supplement to expand MSCs from other tissue sources such as Wharton jelly [126, 170], umbilical cords [171–174], amniotic fluid [175, 176], dental pulp [177–179], periodontal ligaments [180], and others [121]. HPLs are therefore emerging as a universal supplement for in vitro xeno-free expansion of MSCs [12, 121, 181] for clinical transplantation. Studies to identify which types of HPL provide optimal expansion of a given MSC isolate are however recommended [6].

The number of publications describing the use of HPLs for expanding differentiated cells is also growing [121], most studies evaluating whether such xeno-free growth culture conditions can sustain the quality of in vitro-expanded cells for optimal therapeutic outcomes. One focus has been on expanding primary articular chondrocytes intended for treatment of joint alterations, cartilage damage, and osteoarthritis. HPLs stimulate proteoglycan and glycosaminoglycan (GAG) production, as well as the synthesis of collagen type II by chondrocytes and upregulation of SRY-box transcription factor 9 (*SOX9*) gene expression [182]. Better expansion of human chondrocytes was also stimulated by HPLs, although chondrogenic differentiation was more pronounced with FBS [183]. Mixed results were identified in other studies with regards to the capacity of HPLs to support cartilage matrix formation in vitro or to enhance chondrogenesis [121, 184–186]. Recent studies found that HPLs can stimulate the propagation of pediatric auricular cartilage stem/progenitor cells, enhanced the preservation of a spindle-like morphology, with good preservation of surface marker expression, and supported expressions of collagen type II and aggrecan. However, defects in matrix deposition were observed [187]. More systematic studies are deemed necessary to understand the functional effects of HPLs and determine the type and dose of HPLs optimal for chondrocyte expansion. Several studies have consistently pointed out the value of HPLs for xeno-free expansion of human corneal endothelial cells [188–191]. HPL-expanded cells present a good adhesion capacity, hexagonal morphology, and viability, and express vital functional membrane markers such as Na–K ATPase a1, zona occludens-1, phospho-connexin 43, and N-cadherin [188–190, 192]. However, not unexpectedly, not all HPL types are fully equivalent as a supplement for corneal endothelium cells (CEC) expansion. For instance, 1-h treatment of platelet lysate materials at 56 °C was found to enhance the proliferation of CECs [188, 193]. Recent data suggest that HPLs may be of value for safe clinical-grade expansion of dermal fibroblasts for cell therapy and tissue-engineering intended for the treatment of facial scars and burns [194] as well as for the expansion of tenocytes [195].

HPLs also appear to be useful for xeno-free expansion of immune cells like macrophages [196], dendritic cells (DCs) [197], T-lymphocytes, and chimeric antigen receptor (CAR)-T cells [196, 198]. HPL-expanded DCs exhibit good viability, a normal morphology, a satisfactory endocytic capacity, normal phenotypes, and good functional plasticity [197]. They may, however, have a lower type-1 polarization capacity, possibly affecting the suitability to be used as DC vaccines [199], further highlighting that more studies are needed to optimize culture conditions. HPLs can also help in expanding T-cells and CAR-T cells [200–203]. Medium supplementation with one pathogen-reduced allogeneic HPL decreased the doubling-time of a certain type of CAR-T cells [204], increased the percentage of T_CM_ cell subsets, and improved cell functionality, their in vivo antitumor effects, and otherwise overall performance [198, 202, 205, 206]. A pathogen-reduced HPL showed benefits for preparing T cell- and CAR-T cell-based therapies, with a higher proliferative capacity of T-cells and increased CD4 + and CD8 + cell numbers [207]. By decreasing the doubling time of CAR-T cells, HPLs allow cells to replicate more quickly and multiply in greater numbers, thus increasing the treatment efficacy. Additionally, by increasing the percentage of central memory T-cells (T_CM_) cells, which have greater antitumor effects, HPLs may further increase the therapeutic efficacy. Further research into the use of pathogen-reduced allogeneic HPLs will help define how researchers can increase the efficacy of CAR-T cell therapy in cancer treatment, while research to improve the productivity and performance of CAR-T technology may help expand this cell-based therapy to other clinical domains [200], including for treating solid tumors. These data encourage further evaluation of how pathogen-reduced allogeneic HPL culture medium supplementation can help comply with the clinical safety of T-cell and CAR-T cell therapies and also enhance their efficacy and domains of application.

While HPLs demonstrates robust benefits in facilitating clinical-grade expansion of human cells for transplantation, keeping track of the manufacturing attributes and biochemical characteristics of the HPLs is needed to further improve standardization of use and clinical outcomes [6, 11]. More data are needed to confirm that outdated and pathogen-reduced allogeneic PCs can successfully be used to prepare HPLs to efficiently expand differentiated cells and immune cells for transplantation.

## Allogeneic HPLs for regenerative medicine

While many studies have reported the use of PRP as a growth factor-rich surgical adjunct for direct clinical applications in implantology, dermatology, orthopedic surgery, sports medicine, and other fields (see [208–211] for review), only limited preclinical or clinical evaluations of the use of HPLs made from allogeneic PCs have so far been conducted.

### Ocular treatments

The first example of a move from an autologous to an allogeneic product is seen with the preparation of serum eye drops (SEDs) and other eye drops of human origin (EDHOs), including HPLs, for treatment of dry-eye syndrome and other ocular surface disorders [30, 31, 84, 212, 213]. This situation can be explained by the fact that blood establishments producing SEDs realized the limit and logistical constraints of autologous serum production, and the incapacity of frail patients, or those unable to travel to a blood donation or apheresis center, to donate blood [85]. Indeed, relying on autologous serum presents several challenges. First, the collection and processing of blood from individual patients can be difficult due to limitations of donor eligibility, such as poor venous access or underlying health conditions, making blood donation a challenging process. Second, the production of autologous serum requires repeated blood draws from the same patient at regular intervals, which might not be feasible or practical for long-term use. This process can be burdensome and inconvenient for both patients and healthcare providers. Moreover, there may be inherent variability in the composition of autologous serum, including growth factors, cytokines, and other bioactive molecules, depending on the patient’s health condition. This variability in composition may potentially impact the effectiveness of the resulting treatment. Finally, the volume of blood collected from each individual patient is limited, which can impact the overall cost of the production process, including expenses associated with individual patient’s blood processing, monitoring, and customization [31]. These reasons make production from allogenic donations more cost-effective and better standardized and compliant with GMPs [83, 86, 213].

Therefore, a trend was seen towards developing small-pool allogeneic HPLs or serum, including with the inclusion of pathogen-reduction by riboflavin/UV treatment [214], for treatment of ocular surface disorders, but to our knowledge, using outdated PCs as source material has not been reported. Experimental studies demonstrated the possibility of performing S/D virus inactivation treatment of SEDs without substantially affecting their functional properties [215]. Further research can be conducted to establish whether any virally inactivated pooled HPLs from outdated PCs can be a therapeutic alternative for treating ocular surface disorders [31, 216]. Such research will need to consider various aspects to demonstrate feasibility, efficacy, and safety through well-designed preclinical and clinical studies aiming at assessing the effects on corneal epithelial healing, wound closure, inflammation reduction, and an overall improvement in ocular surface health [31, 217], with reference to existing guidelines [218]. Investigating the underlying mechanisms of action of such allogeneic HPLs by elucidating specific bioactive factors, growth factors, potent EVs, and molecular pathways involved [219, 220], and determining potential side-effects can provide insights into the regenerative and anti-inflammatory mechanisms of allogeneic HPL eye drops, and help determine key quality control tests and specifications [31]. Studies should also conduct stratification approaches based on disease severity, etiology, or other relevant factors to unveil patient subpopulations most benefiting from a particular type of allogeneic HPL preparation.

### Neurological disorders

Interestingly, allogeneic, including outdated and pathogen-reduced, HPLs are being investigated in preclinical animal models for treating neurological disorders of the central nervous system (CNS) (see [7, 70] for recent reviews) including neurodegenerative diseases, such as Parkinson’s disease (PD) [221–223], Alzheimer’s disease (AD) [224, 225], amyotrophic lateral sclerosis (ALS) [226], as well as stroke [227]. and traumatic brain injury (TBI) [222, 228–230]. These HPLs are a pleiotropic source of trophic growth factors, such as PDGF, TGF-β, brain derived neurotrophic factor (BDNF), and antioxidant and anti-inflammatory molecules [7, 219, 229]. These factors exert neuroprotective, neuroregenerative, and neurotrophic properties, supporting the survival, growth, and function of neurons in the CNS [7]. Additionally, allogeneic HPLs contain bioactive molecules with anti-inflammatory, anti-oxidative and immunomodulatory effects, which are valuable in combating neuroinflammation and immune dysfunction associated with neurological disorders [7]. By modulating the inflammatory response and immune activity in the CNS, allogeneic HPLs may help reduce inflammation-induced neuronal damage and create a conducive environment for neuronal repair and regeneration [231].

Allogeneic HPLs are also a reservoir of EVs, including exosomes released by platelets during collection and storage processes, or generated during the preparation of the lysate [127]. These vesicles carry a cargo of bioactive molecules, including micro (mi)RNAs, proteins, and lipids, which can be transferred to recipient cells, including neurons [127]. EVs and exosomes play crucial roles in intercellular communication, neuronal survival, and synaptic plasticity. They can modulate disease processes, support neuronal function, cross various brain barriers, and diffuse into the brain, making them likely contributors for therapeutic interventions in neurological disorders. From a practical perspective, allogeneic HPLs can be produced from a pooled source of outdated, potentially pathogen-reduced PCs that are no longer suitable for transfusion [222, 229, 230]. This provides a readily available and standardized source of platelet-derived factors, ensuring consistency and quality in the manufacturing process. The use of outdated allogeneic HPLs simplifies production and reduces the potential constraints and inadequacies associated with using autologous sources from severely ill patients [7]. Moreover, allogeneic HPLs can undergo pathogen-reduction, such as Intercept treatment [230], mild heat treatment [221], and nanofiltration [222] and (b) purification steps [221] to adhere to established safety guidelines and regulatory standards, ensuring the safety and efficacy of the final product. Preclinical developments are on-going to establish the safety and efficacy of dedicated HPLs made from pathogen-reduced allogenic outdated PCs for treatment of ALS [226].

### Other clinical indications

It can be anticipated that the next few years will see additional preclinical and clinical development of applications of HPLs made from allogeneic PCs collected by blood establishments in clinical domains where autologous platelet materials, like PRP or platelet gels, are currently used, such as osteoarthritis [232, 233] or the treatment of recalcitrant wounds [234]. Published clinical reports using allogeneic platelet materials or HPL in the treatment of hip and knee osteoarthritis [235], bone reconstruction [236], or wound healing [237], including diabetic ulcers [238], show the trends towards the development of standardized products for improved consistency of treatment.

## Emerging interest in stand-alone p-EV-based biotherapies

### Scientific and clinical rationale of p-EVs-based biotherapy

Developments seen in the use of allogeneic platelet lysates in regenerative medicine provide scientific and technical rationales for looking at the possibility of using p-EVs, which (a) are abundantly present in HPLs, (b) may contribute to their functionality, and (c) are relatively easy to isolate [74, 127, 239, 240] as stand-alone biotherapy [8]. The relative ease of access to HPLs may indeed provide a source of EVs that is more convenient than other cell types, including MSCs, for which various technology and regulatory challenges still need to be addressed before they can emerge as an abundant, economical therapy [8, 241]. Clinical-grade platelet lysates represent a potentially abundant and complementary source of EVs that could serve specific needs in human regenerative medicine and targeted treatment of diseases [8]. Table 3 summarizes various translational advantages exhibited by p-EVs prepared from allogeneic PC from blood establishments for applications in regenerative medicine and for use as TDDS, as well as various points to consider, as developed below.

**Table 3:** Translational advantages and points to consider about platelet extracellular vesicles (p-EVs) made from allogeneic platelet concentrates (PCs) for regenerative medicine or as drug-delivery system (DDS)

	Features	Comments
Supply	• Allogenic single-donor or pooled PCs collected by blood establishments following GMPs • Autologous PCs obtained from a patient using plateletpheresis collection procedure (or alternatively whole blood collection)	• Allogeneic PCs are a known cellular product on the WHO Model List of Essential Medicines • Platelets are concentrated 3–fivefold in PCs compared to their basal level in the blood circulation, providing a concentrated cellular source for EV production • Outdated PCs, no longer suitable for transfusion, can be a source of p-EVs, therefore not competing with transfusion needs. If needed, allogeneic PC collection dedicated to p-EV preparation is technically feasible • PCs can be used fresh, or alternatively stored frozen and used directly as source material to generate and isolate p-EVs • The lack of ex vivo expansion to generate p-EVs facilitates clinical translation
Pathogen safety	• Donors of allogeneic PCs are screened and donations are tested by serological and/or NAT to limit the risks of TTI (e.g., HIV, HBV, and HCV) • PCs can be subjected to licensed photochemical treatments to inactivate most bacteria, viruses, and parasites	• The collection process and storage of PCs are conducted under aseptic conditions using dedicated licensed single-use medical devices. There is a residual incidence of bacterial contamination of approximately 1 in 2000 in PCs occurring during venipuncture • Risks of viral contamination (due to window phase donations of known and tested viruses or emerging, untested, viruses) cannot be excluded. The risk is approximately 1 in 1–2 million when PCs are collected from healthy donors in countries with a regulated blood system • p-EVs made from pools of multiple donations should preferably be prepared from pathogen-reduced PCs • The lack of a nucleus in platelets makes it feasible to use photochemical treatments designed to alter nucleic acids and inactivate blood-borne pathogens
Immunological safety	• The PC material should be tested for the presence of platelet antigens to avoid alloimmunization by recipients	• Platelets express antigens (e.g., ABO, HLA class 1, or HPA antigens) that can cause alloimmunization in incompatible recipients. These antigens may be present on p-EVs and may potentially be clinically significant • Leukoreduction of PCs decreases risks of contamination of p-EV preparations by leukocytes expressing HLA class I and HLA class II
Procoagulant activity	• PCs are collected in the presence of a citrate anticoagulant solution and stored for up to 5 to 7 days following licensed procedures intended to minimize platelet activation	• Generation methods of p-EVs may generate p-EVs with exacerbated procoagulant activity. Process validation and quality control tests can be used to check the prothrombogenic activity of p-EVs

*HLA* human leukocyte antigen, *HPA* human platelet antigen, *NRA* national regulatory authority, *TTI* transfusion-transmitted infection, *WHO* World Health Organization, *LMICs* low- and middle-income countries, *NAT* nucleic acid testing, *HIV* human immunodeficiency virus, *HBV* hepatitis B virus, *HCV* hepatitis C virus, *GMPs* good manufacturing practices

Briefly, allogeneic PCs provide a readily available and abundant source for the production of p-EVs, facilitating large-scale production and the development of standardized manufacturing processes without considering additional specific production and regulatory issues (a GMP facility, compliance and suitability of growth medium) associated with obtaining EVs as byproducts of cell cultures [242, 243]. Using allogeneic PCs collected using already approved standardized protocols can provide better control over the composition and quality of p-EVs. The fact that blood donors are screened, and donations are tested and potentially undergo pathogen-reduction steps contribute to ensuring the safety of derived p-EVs. Finally, the existing infrastructure for the preparation of PCs, based on the nearly 120 million blood donations collected each year at the global level [244], provides an abundant possible supply for scalability, optimal cost-effectiveness, and potential widespread clinical translation using local platelet resources [8].

However, points to consider include the fact that little is still known about the impacts of donor variations and processing in terms of cargo (miRNAs, proteins, and lipids), potency, and therapeutic effects of p-EVs. There is indeed a possibility that p-EV compositions vary depending on donor characteristics, the platelet activation status, and manufacturing processes [8]. Identifying and characterizing specific cargoes and their functional implications are important for targeted applications. It is also essential, as for any type of EV, to develop, validate, and carefully monitor standardized protocols for the isolation, purification, characterization, and safety testing of allogeneic p-EVs [8, 74, 240]. Meeting compliance with regulatory guidelines and obtaining appropriate approvals for the use of allogeneic p-EVs in regenerative medicine or as TDDSs will also be necessary [8]. Preclinical and clinical studies, most preferably conducted following double-blind placebo-controlled protocols [245], will be required to evaluate the efficacy and safety of allogeneic p-EVs and obtain marketing authorization. Aspects to evaluate, among others, should include demonstrated therapeutic potential, dose and frequency of administration, administration routes, and potential adverse effects. Recently, initial results of a double-blind placebo-controlled clinical study of allogeneic small-pool p-EVs for wound healing were reported, demonstrating feasibility of this approach [246]. Such allogeneic p-EV, manufactured from PC collected from healthy donors, have the potential to emerge as an effective and standardized biotherapy [8].

### Source material, generation, and purification of p-EVs

Blood, from which PCs and HPLs are prepared, contains up to 10^8^–10^9^ EVs/µl [46, 247] under normal physiological conditions, with platelets and megakaryocytes jointly contributing to close to 50% of the EV blood pool [72, 248, 249]. It should also be kept in mind, however, that blood is a very complex fluid, and determining the total number of EVs in PCs and HPLs is challenging due to possible co-isolation of lipoprotein particles [250]. PCs contain approximately (2–5) × 10^9^ platelets/ml, and their supernatants can yield about 10^11^–10^12^ “EV-like” events/ml depending upon the methods used for EV generation [74, 251–253] and the assessment method [133, 254–257]. Studies have shown that the mean size of most EVs in plasma and serum is in the 100–300-nm range [47, 258]. It is expected that EVs present in PCs have diverse cellular origins, representing the EV population in the donor’s blood at the time of blood collection [72, 247, 248], being derived from erythrocytes, WBCs, platelets, megakaryocytes, or vascular endothelial cells. This naturally occurring population of EVs is likely enriched by p-EVs released by platelets during PC collection, processing, and storage. Several processing steps performed during the preparation of PCs, such as whole blood collection versus apheresis, leukoreduction, pathogen reduction, and the duration of storage at 20–24 °C under agitation, may also influence the total number and type of EVs present in PCs [133, 255, 259]. Systematic studies to delineate the impacts of PC processing on the number and functionality of PC-EVs are thus still needed.

HPLs obtained by various processing methods, such as freeze–thaw or calcium chloride activation, were found to contain approximately 10^12^ EV-like nanoparticles/mL [127]. Two main types of EVs are thought to occur in HPLs, namely microvesicles and exosomes [260], but this is likely an over-simplification, and more research is needed to understand the complexity of p-EV populations. Microvesicles, with sizes ranging 100–300 nm and larger, are shed from plasma membranes [242], while exosomes, or small EVs, with a diameter of 30–100 nm, originate from endosomal multivesicular bodies and alpha-granules [239]. The zeta potential of p-EVs is slightly negative (-5 mV or less) [127], similar to values reported for EVs from other cell types [261]. Several treatments can be used to trigger the release of EVs from platelets present in PCs. These methods can be applied to whole PCs themselves (platelets suspended in plasma or plasma/PAS), or on platelets isolated from PCs by a centrifugation step at ca. 3000 × *g* for 20 min at 20–24 °C [8, 80, 127, 240]. Physico-mechanical methods, such as freeze–thaw cycles, shaking, and sonication [262] can be applied to disrupt platelet membranes leading to EV release. Such methods have been reported to lead to the release of as many as 100 to 500 p-EV (or p-EV-like events) per platelet [262]. Alternatively, chemical and biochemical methods involving incubation with Ca^2+^ ionophores (to activate the coagulation cascade), lipopolysaccharides, thrombin, collagen, or ADP, to activate platelets through binding to their receptors, can also stimulate the release of p-EVs [74, 263]. The ways that PCs are collected and HPLs prepared impact the type and proportions of EVs expressing different markers relative to CD41a, specific to p-EVs, confirming a diversity in p-EV populations [264]. Much work is thus needed to better characterize the nature of EV populations released by platelets.

Several procedures have been proposed for purifying EVs from PCs or from isolated platelets, including differential centrifugation, ultracentrifugation, and size-exclusion chromatography, as for other EV types [8, 74, 127, 240, 265, 266]. The most common methods used so far combine low (ca. 300 × *g*) and intermediate (ca. 3000 × *g*) speed centrifugation, to remove cell debris, with 20,000–100,000 × *g* ultracentrifugation to pelletize p-EVs, exploiting differences in size and density between cellular components and debris, protein components, and EVs to achieve their partitioning. The obtained EV pellet can be resuspended in a suitable buffer for further and final processing, formulation, bacterial filtration, and dispensing into the final container. The selection of an optimal isolation method may vary based on the sample source, the downstream specifications of purity, and the intended applications. For instance, some studies used size-exclusion chromatography with resins, such as Sepharose CL-2B [127, 264, 267], with a porosity of 35–75 nm to remove proteins and isolate EVs based on their size and shape, rather than their density. Defining an optimal method to produce p-EVs for clinical applications should therefore consider various factors such as the impacts of blood-collection procedures and mode of preparation of HPLs, process efficiencies, p-EV sub-population characterization, impacts on the downstream process, and complexity of regulatory approval for clinical evaluation [8]. Producing p-EVs from pools of allogeneic PCs or platelet lysates can be a powerful means to enhance the consistency of p-EV properties [8]. This, however, needs to address also other challenges such as guaranteeing pathogen safety.

### Pathogen safety of p-EVs

Ensuring the virus safety of p-EVs derived from allogeneic PCs overlaps partially that needed for HPL, and is essential for clinical translation, as summarized in Table 4. It is vital that only allogeneic PCs meeting quality specifications for transfusion are used to prepare clinical-grade p-EVs. However, several virus-reduction treatments that provide robust safety for plasma protein products and HPLs, such as dissolution of lipid envelopes by S/D, or removal by 20-nm nanofiltration [34, 160, 163, 222], are not seen to be applicable to EV preparation. This is due to common structural features shared between EVs and viruses, including the presence of a lipid membrane and a similarity in nanometer size. Pathogen safety of allogeneic p-EVs should therefore currently be best assured by donors’ screening, donation testing, and pathogen-reduction treatment of the starting PC materials using photochemical or photoinactivation procedures [104, 113]. It is important to note that these methods do not guarantee protection against all viral risks [109], a concern for large-pool EV products that would not be subjected to one additional virus-reduction step. In addition, some experimental data suggest that photoinactivation treatments of PCs may affect the miRNA content [133, 268] but how this can influence the regenerative and protective functions of p-EVs is still unknown. Therefore, further research should be conducted to develop novel pathogen-reduction procedures that minimally affect p-EV functionality. Until such technological developments are achieved, it is crucial to rely on screened donors and clinical-grade donations that undergo rigorous screening and testing following international guidelines [33] to respectively ensure donor eligibility and detect potential infectious diseases. This is vital to help minimize the risk of transmitting viral infections through p-EV preparations especially when derived from pooled donations.

**Table 4:** Examples of quality and safety requirements of allogeneic platelet lysates and platelet extracellular vesicles (EVs; p-EVs) as medicinal preparations

	Platelet lysates	Platelet extracellular vesicles
Blood donors	Meet health requirements for donations of whole blood or platelet concentrates (PCs)	Meet health requirements for donations of whole blood or PCs
Whole blood or platelet concentrate donations	Meet overall quality and safety requirements for clinical use: testing of viral markers, blood cell counts within specifications	Meet overall quality and safety requirements clinical use: testing of viral markers, blood cell counts within specifications
Pathogen-reduction treatment of individual donations	Licensed pathogen-reduction treatment of PCs (e.g., photochemical treatment)	Pathogen-reduction treatment of PCs (e.g., photochemical treatment) demonstrated to not affect EV functional activities
Treatment of PCs	Freezing, thawing, and pooling	Freezing, thawing, and pooling
Optional processing steps	Fibrinogen conversion (e.g., CaCl_2_ activation); dedicated virus-reduction treatment (e.g., gamma-irradiation; electron-beam; solvent-detergent), low-speed centrifugation; depth filtration; bacterial filtration; aseptic filling; freezing	EV generation from PCs (freeze–thaw, sonication, CaCl_2_ activation); low-speed centrifugation; ultracentrifugation; size exclusion chromatography; bacterial filtration; aseptic filling; freezing
Quality control	Meet specifications: protein content; microbial sterility, endotoxins; pH; osmolality; representative growth factor content (e.g., PDGF-AB); functional assay (e.g., cell proliferation test)	Meet specifications: protein content; EV number; representative p-EV markers; microbial sterility; endotoxins; pH; osmolality; representative growth factor content (e.g., PDGF-AB); functional assay (e.g., cell proliferation test; internalization assay)

*PDGF* platelet-derived growth factor

### p-EVs as regenerative medicine biotherapy

Recently, the concept of p-EVs as a key effector of the regenerative activity of allogeneic HPLs has emerged, leading to mounting interest in evaluating p-EVs as a stand-alone clinical adjunct in various fields of regenerative medicine [8, 246]. Thanks to paracrine action associated with the broad repertoire of trophic factors they carry and transfer to recipient cells, p-EVs are capable, like MSC-EVs [269], of modulating biological pathways of tissues by directly activating target cells, homing in on injured or inflamed tissues [270] where they help maintain stem cell populations and prolong their lifespan in vitro [239], and inducing the release of biomolecules by neighboring cells [8, 271–276]. Thus, p-EVs may be capable of modulating the mechanisms of ageing-associated diseases [8, 242, 249, 277]. Functional molecules present in p-EVs encompass various growth factors, chemokines, cytokines, pro- and anti-inflammatory factors, pro- and antiangiogenic factors, pro- and anticoagulants, antioxidants, matrix metalloproteinases originally stored on platelet granules, in addition to lipids and nucleic acids (messenger (m)RNA and miRNA) [47, 73, 74, 220, 278–280]. Therefore, p-EVs may play critical roles in tissue regeneration by providing a wide range of synergistic biomolecules to promote cell proliferation, migration, differentiation, angiogenesis, and remodeling of the extracellular matrix and regulating gene expressions [8, 281–285].

For instance, VEGF, PDGF, and TGF-β released by p-EVs may promote cell proliferation, migration, and differentiation of fibroblasts, endothelial cells, and MSCs at the site of injury, as well as promoting the formation of new blood vessels to bring oxygen and nutrients to sites of injury as shown in a rat model [286]. Lipid components of p-EVs also contributed to tube formation by human umbilical vein endothelial cells (HUVECs) [287], and a proangiogenic effect, attributed to VEGF, b-FGF, and PDGF, was found in a rat model, allowing for better revascularization following chronic ischemia [286]. p-EVs isolated from HPLs contain miRNAs, including miRNA126, which can modulate expressions of genes involved in cellular mechanisms important for angiogenesis in a HUVEC model [280]. In an animal model of trauma, p-EVs contributed to maintaining hemodynamic stability and mitigated the development of ischemia and metabolic acidosis [288].

Recently, p-EVs were found to help heal wounds in diabetic rat models [289], repair endothelial corneal defects [220], treat intervertebral disc degeneration [290] and damaged tendons [273, 280] thanks in part to their inherent anti-inflammatory and antioxidative properties. Interestingly, p-EVs, rich in neurotrophic factors (e.g., PDGF, BDNF, and b-FGF), neurotransmitters, anti-inflammatory factors, and antioxidants [7, 220], may contribute to the emerging platelet-derived neuroprotective biotherapy of neurodegenerative disorders by promoting neuronal survival and reducing neuroinflammation [7]. Administration of p-EVs by topical/intracranial routes allowed their diffusion into the brain and stimulated neurogenesis and improved behavior in a stroke model [291], stimulated the differentiation of neural stem cells into glia and neurons [292], and generally promoted neurogenesis [221, 293, 294]. One p-EV population isolated from serum-converted HPLs by size exclusion chromatography promoted network formation in primary cortical neuronal cultures [127]. In addition, some EV subsets contain functionally active mitochondria [72, 254], supporting possible translation to treatment of mitochondrial abnormalities and the recovery of cell functions.

Identifying key molecular players will help the clinical development of p-EVs, but much work is still needed [295, 296]. In addition, to date, most of the assumptions as to possible applications of p-EVs in regenerative medicine are still relatively scarce and rely on laboratory-scale experiments using “homemade” preparations. Translational work is needed using p-EV preparations made from pooled allogeneic HPLs processed under scalable clinical-grade conditions eventually capable of meeting regulatory requirements. Also, one needs to understand how the methodology of p-EV generation and isolation may impact surface markers and cargo, and thus their safety and functionality [8, 74, 240]. Additionally, a comparative assessment of the functionality of p-EVs in relation to various MSC-EVs, considering their physiological cargo, would be highly interesting to identify their respective therapeutic values.

### p-EVs as a targeted drug-delivery system (TDDS)

The rationale for using p-EVs as a TDDS is a logical progression of previous studies which demonstrated that platelets themselves are potent vehicles for drug delivery, especially in the context of cancer treatment [80, 242, 297–305] and immune and inflammatory diseases [242, 301, 302]. p-EVs' value as a targeted drug-delivery vehicle to cells and tissues is also linked to the specific range of surface markers originating from platelet parent cells. Due to their nanometer size, p-EVs are expected to have a better capacity to reach and be retained by tumors [80, 306], and at sites of inflammation such as with pneumonia [307].

#### P-EVs membrane markers important for tissue targeting

p-EVs are characterized by expressions of various glycoproteins/clusters of differentiation (CDs) originating from platelets that contribute to cellular targeting and cross-talk with pathological sites [308]. Instrumental p-EV membrane markers include CD41, also known as integrin αIIb, and CD61 (integrin β3) which together form the platelet-specific integrin alpha-IIb/beta-3 (GPIIb/IIIa) that plays crucial roles in platelet aggregation, adhesion, and cell targeting and retention. CD42 (glycoprotein Ib or GPIb) is a receptor for vWF and contributes to p-EVs' adhesive properties and interactions with specific cell types or endothelial surfaces. CD62P (P-selectin), a marker of platelet activation, is a cell adhesion molecule that interacts with the P-selectin glycoprotein ligand (PSGL)-1 on leukocytes during inflammation (e.g., atherosclerosis), immune responses, and thromboses, contributing to the recruitment and retention of p-EVs to pathological sites. Platelet endothelial cell adhesion molecule-1 (PECAM-1; CD31) plays a role in cell–cell adhesion, contributing to the adhesion and transmigration of platelets across endothelial cells during inflammation. Thus PECAM-1 may trigger p-EVs to adhere to the endothelium and migrate into underlying tissues [309]. These membrane markers are instrumental in cellular targeting [281, 283, 288] and in cross-talk with pathological sites [80, 310], and can thus facilitate the adhesion of p-EVs to specific cell types or surfaces and enable the delivery of cargoes, but more research is needed to elucidate how p-EV generation procedures impact expression levels of these markers.

#### Loading of drugs within p-EVs

The loading of hydrophobic and hydrophilic drugs can be achieved by various methods [8, 75, 242, 311]. In passive loading, isolated p-EVs are incubated with drugs, allowing them to enter through diffusion or a concentration gradient, taking advantage of the natural permeability of the lipid bilayer of p-EVs [262]. In active loading, methods such as electroporation, sonication, and extrusion are employed to disrupt the lipid bilayer of p-EVs, and create transient pores or channels through which drugs can be actively loaded to achieve higher encapsulation efficiencies [242, 262, 265, 266]. Pre-loading drugs into platelets, before the isolation of p-EVs, is another option. Platelets can be loaded with drugs via a combination of incubation, electroporation, freeze-thawing, calcium chloride activation, and sonication, with loaded platelets being activated to release drug-loaded p-EVs [8]. Removal of the unloaded drug typically involves processes based on size exclusion to segregate loaded p-EVs from the free drug [262]. An important element to consider for industrial and economic feasibilities is the yield of production of p-EVs from platelets. Recent studies showed that sonication and extrusion respectively generate means of 496 and 493 p-EVs per platelet, more than the 145 p-EVs by freeze/thawing and 33 p-EVs by 37 °C incubation [262]. The method by which p-EVs are generated and loaded can have detectable impacts on the structure and safety of p-EVs. For instance, recent data suggested that based on the level of expression of PS, p-EVs obtained by freeze-thawing of platelets would be less prothrombogenic than those generated by extrusion or sonication [75]. However, more studies are needed to delineate the impacts of the generation and drug loading procedures on p-EV procoagulant activity for any desired therapeutic application. Once loaded, the drugs are protected from degradation and are expected to be released slowly over time at the site of injury, ensuring sustained drug delivery.

#### Clinical developments of p-EVs as DDS

Clinical developments in the use of p-EVs as TDDSs are still limited. One expected area of clinical translation of drug-loaded p-EVs is for the delivery of anticancer agents based on the known pathogenic loop existing between tumors, platelets, and p-EVs [80, 281, 312–315] and a capacity for p-EVs to be internalized by cancer cells [80]. This expectation was confirmed by a recent study showing that drug-loaded p-EVs could be internalized and exhibited higher cytotoxicity than free drug in vitro against cancer cell lines [262] as well as in clinical samples from leukemia patients [305]. Other pathological targets are atherosclerosis and other diseases associated with inflammatory processes [75]. For instance, in an experimental model, p-EVs loaded with an NLRP3-inflammasome inhibitor allowed significant reduction in atherosclerotic plaques, a decrease in local inflammation and macrophages and T-cell proliferation at plaque sites in ApoE-knockout (KO) mice [316]. Similarly, p-EVs loaded with the [5-(*p*-fluorophenyl)-2-ureido]thiophene-3-carboxamide (TPCA-1) anti-inflammatory molecule could target pneumonia in a mouse model of acute lung injury, inhibiting expressions of inflammatory factors and the infiltration of pulmonary inflammatory cells, and making it possible to modulate cytokine storms [307].

#### Future prospects in using p-EVs as TDDS

Although still limited, these experimental data support the potential for p-EVs for use as TDDSs in various clinical applications [8]. p-EVs appear to be particularly potent vehicles to deliver their therapeutic payloads specifically to cancer cells [262, 317], with the potential to improve retention within the tumor microenvironment (TME), minimize off-target effects, and reduce systemic toxicity [8, 75, 317]. It was speculated that in inflammatory and autoimmune diseases involving a functional role a platelets, p-EVs could be used to deliver immunosuppressive agents or anti-inflammatory drugs to sites of inflammation or autoimmune activity, potentially improving the treatment of conditions like rheumatoid arthritis, inflammatory bowel disease, and atherosclerosis [8, 318]. In cardiovascular diseases, p-EVs can interact with endothelial cells, promote angiogenesis, and modulate cardiovascular functions. They can be utilized for targeted delivery of therapeutics to promote cardiac repair, neovascularization, and tissue regeneration, or modulate oxidative stress after myocardial infarction or peripheral artery disease [75, 319]. In neurological disorders, p-EVs, similar to other nanoformulations, can be loaded with neuroprotective or neuroregenerative factors, as well as antioxidants and targeted to specific cell types in the CNS, offering a promising avenue for treating conditions like neurodegenerative diseases, stroke, and TBIs [7, 320]. While the use of p-EVs as TDDSs appears safe and promising, additional preclinical studies followed by rigorous clinical trials, as recommended for “naïve” p-EVs [245], will be necessary to establish their efficacy, optimize their delivery strategies, and ensure their safety in various clinical applications such as those listed here [8]. In summary, compared to other DDSs, allogeneic p-EVs offer several practical and functional advantages over other types of EVs or DDSs, including (a) the ease of access to a standardized platelet source material, (b) a human origin that confers good biocompatibility and low immunogenicity, and (c) their natural targeting capabilities due to the presence of platelet membrane receptors and surface proteins making it possible for p-EVs to interact with specific target cells or tissues.

### Lessons learned from the use of platelet membranes to prepare “p-EV-like” nanomedicines

Recently, there is a research trend in using platelet membrane coating to enhance the biocompatibility, residence time, targeting, and functional capacity of p-EV-like nanomedicines for applications in the treatment of various inflammatory diseases, including atherosclerosis, thrombosis or cancer [321–324]. Platelet membrane coating was shown in experimental models to be of special value to enhance the adhesion to the sub-endothelium, provides immune evasion, and interactions with pathogens, and, in general terms, improved homing and retention at the pathological sites for optimal drug release [324, 325].

Among other examples, one promising area of clinical development is the use of such platelet cloaking approach to treat myocardial infarction (MI), especially in a context where the heart shows very low uptake of therapeutics after MI [326]. The rationale lies on the fact that platelets play important biological roles in MI injury and repair. Firstly, platelets accumulate at the MI injury site, particularly at injured endothelial cells via binding to vWF using surface glycoproteins including GPIb, GPIIb, GPVI, GPIV [327]. Thereupon, they release cargo which modulates inflammation and immune cell recruitment. In addition, platelets have been shown to bind circulating CD34 + progenitor cells and macrophages, which are, in-turn, recruited to the injured heart [328]. Thus, using platelet membrane is a rational approach for use as drug delivery systems in MI. It was indeed shown that liposomes functionalized with platelet membranes expressing GPIIb and CD42c (GPIb-beta) were able to bind to circulating monocytes and could be recruited to the MI site in greater concentrations, and for prolonged periods of time [329]. The liposomes were used to carry an anti-inflammatory small molecule Cobalt protoporphyrin IX (CoPP), which reduced MI severity and improved cardiac function in a mouse model. Platelet “nanovesicles” could be used to enhance the delivery and retention of cardiosphere-derived stem cells (CSCs) by altering CSC membrane signatures [330]. These CSCs showed improved engraftment and resulted in better therapeutic outcomes in both rodent and porcine models of MI, compared to unmodified CSCs.

Another study modified MSC-derived EVs with platelet membranes to achieve improved endothelial cell targeting and angiogenesis in a mouse model of MI [331]. Modification of MSC -exosomes with platelet membranes reduced their uptake by macrophages, enhanced targeting to the injured heart and promoted micropinocytosis, resulting in enhanced cellular uptake by endothelial cells and cardiomyocytes. This resulted in improved cardiac functions in a mouse model of myocardial infarction injury [332]. Lastly, platelet membranes were used to functionalize PLGA nanoparticles carrying miRNA inhibitors [333], reducing infarct size and improving cardiac function in a rat MI model.

Therefore, platelet membrane coating of nanomedicines may offer several synergistic advantages [8, 75]. In brief, the enhanced biocompatibility can reduce the risk of immune recognition, clearance, and adverse reactions. Prolonged residence times can allow enhanced accumulation at the target site and improve the potential therapeutic efficacy. The presence of platelet membrane markers can improve the targeting ability to pathological sites or cell types, thereby enhancing internalization by target cells, improving the therapeutic potential and reducing off-target effects [323, 324, 334]. Altogether this field of preclinical research supports the scientific rationale towards the development of p-EVs as stand-alone carriers for TDDSs.

Platelet membrane-coated nanomedicines hold potential in human medicine but some possible trends can be considered to further improve the therapeutic value and domains of application, as well as production capacity. For instance, incorporation of additional ligands or functional molecules onto the platelet membrane surface may further enhance targeting specificity, synergistic effects, and multifunctionality for personalized medicine approaches of drug-loaded nanocarriers. While cancer therapy has been a primary focus, it may be valuable to expand the scope and impact of platelet membrane-coated nanomedicines to other areas, such as cardiovascular diseases, neurodegenerative disorders, and inflammatory conditions. Last but not least, manufacturing techniques should be refined to make it feasible to prepare platelet membranes under conditions meeting GMPs and allowing scalability.

### Possible thrombotic risks associated with p-EVs

p-EVs are promising therapies, but one safety concern in using them in the clinic is their potential capacity to promote blood clotting. Some populations of p-EVs have a membrane 50–100-fold more procoagulant than that of activated platelets due to expressions of binding sites for activated factor V and factor VIII, and thrombin [335] or exposure of negatively charged PS and P-selectin (CD62P) [284, 336, 337]. Therefore, some p-EVs may increase the risk of thrombotic events, which is of specific concern for patients with pathologies like cancer associated with a procoagulant state. The extensive experience and safety testing developed in the human blood product industry may provide valuable insights and methodologies that can be applied to assess the quality and safety of p-EVs, particularly in relation to the risks of pro-coagulation and thrombogenicity. Several assays can be considered, including the thrombin generation assay (TGA) that measures the in vitro generation of thrombin [338] and has proven useful when adopted to predict the procoagulant potential of intravenous immunoglobulin preparations [339]. The TGA provides information about the potential procoagulant activity associated with p-EVs and their ability to initiate clot formation by documenting various parameters, such as the lag time, peak thrombin concentration, and endogenous thrombin potential, to evaluate the thrombotic potential of p-EVs [340, 341]. This assay can be used in conjunction with other tests to determine the exposure of PS and TF, including various clotting time assays, activated partial thromboplastin time (aPTT), prothrombin time (PT), phospholipid-dependent clot-based assays, and determination of platelet activation markers, such as CD62P [125, 342–345]. Animal models, such as venous or arterial thrombosis models, can be also be considered as preclinical models to assess the thrombotic potential of p-EVs by evaluating possible induced thrombus formation as has been done for some plasma products [346].

Another concern is a possible excessive proinflammatory effect of some p-EVs due to their content of proinflammatory cytokines like interleukin (IL)-1, IL-6, and TNF [347]. However, studies have observed that some p-EV populations are capable of suppressing inflammation through inhibiting the release of TNF by macrophages [348], and the release of TNF and IL-8 by plasmacytoid DCs [349]. Differences were found in the capacity of two p-EV populations to aggregate monocytes and induce NETs [336, 350]. Thus, p-EVs may play differential roles in triggering inflammation. As for any complex biotherapy, little is still known about any dose-dependent effects, which implies that the effects of treatment may vary depending on the dose used and the frequency of administration. This can make it challenging to determine the optimal safe and efficacious treatment within the scope of a specialized individual medicine. The long-term effects of p-EV therapy, as with any treatment based on EVs, are largely unknown, thereby highlighting the need for more preclinical research to understand their long-term safety and efficacy. As for any biologicals and EVs from other cellular sources [242], process validation and monitoring, as well as in-process and final product quality control and standardization in the preparation and characterization of p-EVs are vital to support consistency in quality, safety, and efficacy [8].

### Future trends in p-EV-based biotherapies

**Fig. 4:**
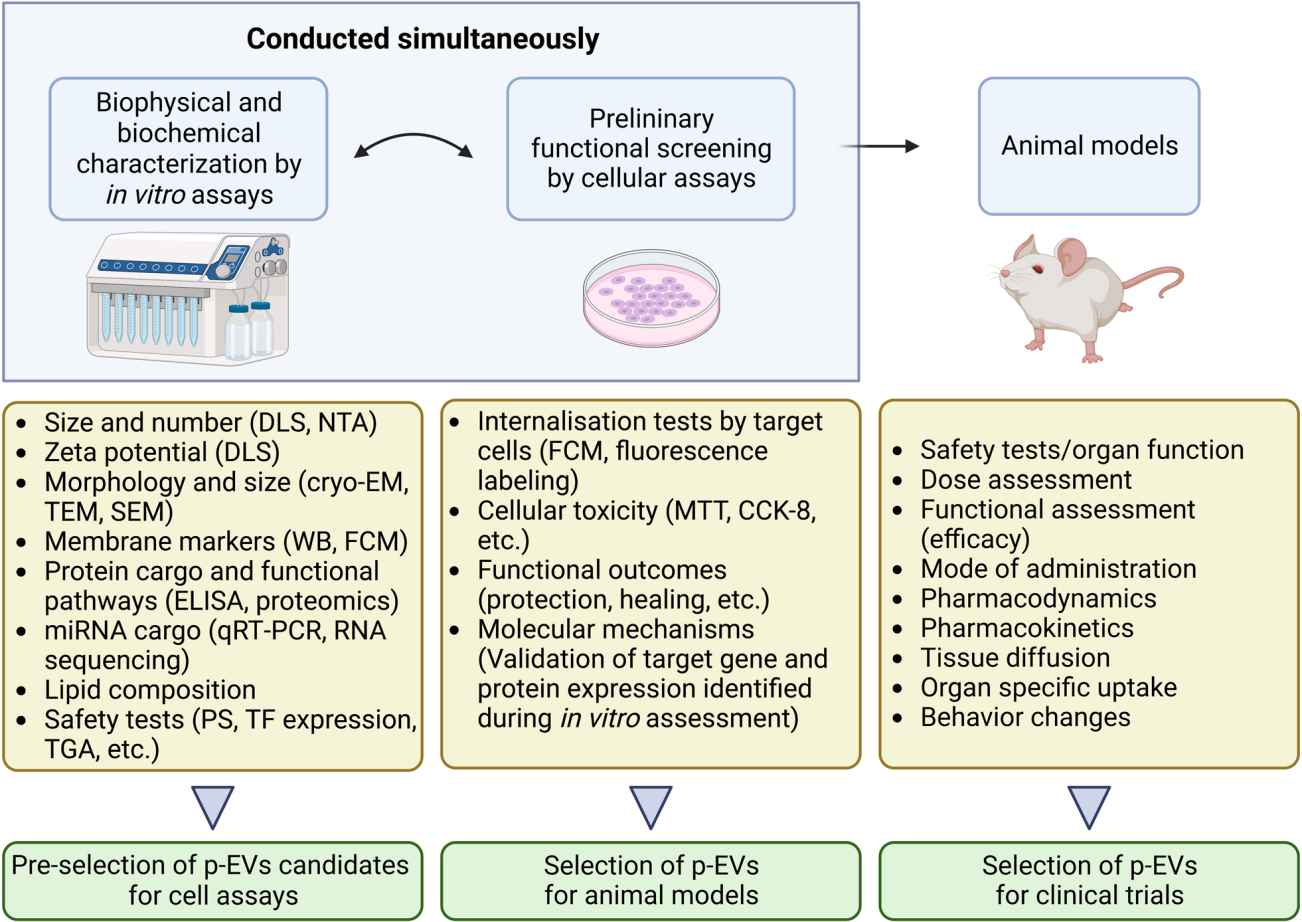
Overview of a range of in vitro, cellular, and animal preclinical tests that can be conducted to characterize and select the optimal populations of platelet extracellular for clinical translation. *DLS* dynamic light scattering, *ELISA* enzyme-linked immunoassay, *CCK-8* cell counting kit-8, *EM* electron microscopy, *p-EVs* platelet extracellular vesicles, *FCM* flow cytometry, *MTT* 3-(4,5-dimethylthiazol-2-yl)-2,5-diphenyl tetrazolium bromide, *NTA* nanoparticle tracking analysis, *PS* phosphatidyl serine, *PC* platelet concentrate, *qRT-PCR* quantitative reverse phase polymerase chain reaction, *RNA* ribonucleic acid, *TGA* thrombin generation assay, *TF* tissue factor, *WB* western blotting

Further research is needed to better delineate the functionality and optimal clinical applications of each type of allogeneic p-EV, also recognizing the advantages and potential constraints of this material. This is important as, compared to MSC-EVs, the use of allogeneic p-EVs has so far received relatively little attention, and no comparative assessments of safety and efficacy have been conducted to our knowledge. As is the case for HPLs, the use of clinical-grade blood donations as a source material for p-EVs has major advantages for clinical translation and to establish proof-of-concept of clinical safety and efficacy of EV therapy. Moving p-EVs towards clinical use can be facilitated when targeting clinical indications where the benefits of platelet biomaterials, themselves rich in p-EVs, have been demonstrated and where the presence of p-EVs has been shown to contribute to functional outcomes. In addition, rigorous preclinical methodologies should be followed to define the biophysical characteristics, safety, functionality, and optimal clinical applications of each type of purified allogeneic p-EV as summarized in Fig. 4.

Various in vitro biophysical and biochemical assays can be performed, as for any type of EVs, to ascertain the size, number, zeta potential, membrane marker expression, cargo composition (e.g., proteomic and miRNA contents), and possible procoagulant activity as a means to identify populations providing the optimal quality and safety profiles to best meet the intended clinical application [8, 74, 127, 220, 240, 242, 262, 279]. In vitro cell culture studies are invaluable in assessing the toxicity and the functional effects of different types of allogeneic p-EVs on various cell types or disease models, and comparing the capacity for cellular internalization, cellular responses, functional outcomes, and molecular mechanisms to understand specific functions and therapeutic potential [127, 220]. In case good outcomes are seen in cellular models, animal models could help to investigate the therapeutic efficacy and safety profiles of selected allogeneic p-EVs in vivo. These studies can be instrumental in assessing and comparing the regenerative and protective potential, immune responses, biodistribution, and therapeutic outcomes associated with specific p-EV preparations and determine, based on scientifically based and objective criteria, the most suitable allogeneic p-EV types for specific disease conditions. These could be further validated by well-designed clinical trials as crucial instruments for evaluating the efficacy, safety, and optimal clinical applications of allogeneic p-EVs and for monitoring clinical outcomes, disease progression, biomarker analyses, and adverse events in an objective scientifically based manner [245].

One possible approach to target clinical domains where p-EVs could be clinically beneficial is to scrutinize clinical indications where the benefits of platelet biomaterials, such as HPLs containing p-EVs, have been demonstrated. This includes treating ocular surface disorders such as dry eye syndrome, corneal ulcers, and ocular surface injuries, where EDHO and HPL rich in p-EVs, showed promising experimental and clinical results [29–31, 83, 86, 213, 215, 217] in promoting corneal epithelialization, reducing inflammation, and improving overall ocular surface health. Another clinical field is treating chronic wounds where various platelet biomaterials containing p-EVs have demonstrated efficacy in promoting healing, including diabetic foot ulcers and venous ulcers. This approach was confirmed by the fact that the first clinical trial of p-EVs was conducted in wound healing [246]. Similarly, treating osteoarthritis with p-EVs is expected to be evaluated in the near future, as this is one main indication of PRP and now, increasingly, allogeneic HPLs [19, 24, 351]. Finally, interest is emerging in evaluating p-EVs for treating various neurological disorders [127], based on exciting preclinical data showing preclinical benefits of HPLs for brain administration in various neurodegenerative diseases and trauma [7, 70].

### Ethical considerations in using clinical-grade allogeneic PCs as source material for p-EVs

The use of clinical-grade blood or platelet donations as a source material for p-EVs therapeutics should fall within international ethical principles regulating the preparation of current therapeutic blood products, both cell-based and protein-based. These ethical principles are defined in several resolutions adopted by the World Health Assembly (WHA) [352, 353]. In particular WHA58.13 supports “the full implementation of well-organized, nationally coordinated, and sustainable blood programs with appropriate regulatory systems”, and stresses the role of voluntary non-remunerated blood donors from low-risk populations [354]. WHA resolution 63.12 supports efforts to ensure the availability, safety, and quality of blood products in response to major unmet public health needs. These resolutions have been translated into guidelines and regulations published by the WHO, indicating that blood and platelet donors should donate in blood establishments licensed and regularly inspected by national regulatory authorities to ascertain operations following GMPs [33]. Donations should be voluntary and non-remunerated (which according to the WHA should be the target for all countries), to avoid recruiting donors among at-risk populations. Donors should provide informed consent (e.g., by signing a donation information leaflet) for the use of their donations in research or therapeutic applications, and this should apply when donations are used to isolate p-EVs. Donors should be fully informed about the purpose, potential benefits, and possible risks (including infectious risks) of using their donated blood for p-EV production, as any other blood products, so they can refrain from donating if they become aware of a risk factor. The confidentiality and privacy of donor information should be preserved, and measures should be in place to protect donor identities, medical records, and any other sensitive information related to the use of their blood donations. However, full traceability should be in place to make it possible “to trace each individual unit of blood or blood component derived thereof from the donor to its final destination, whether this is a recipient, one or more batches of medicinal product or disposal. The term is used to describe forward tracing (donation to disposition) and reverse tracing (disposition to donation)” in a way similar to that which prevails for plasma utilized for fractionation [355]. Such procedures can allow to perform a “lookback” if it was found retrospectively that a donation from a high-risk donor should have been excluded from processing [355]. At the preclinical and clinical trial stages, collaborating with institutional review boards (IRBs), ethics committees, and regulatory authorities can ensure adherence to established ethical guidelines. In conclusion, the ethical framework already in place for currently marketed cellular and protein blood products should be readily applicable to the production of p-EVs from allogeneic PCs. In addition, the possibility of using outdated PCs not suitable for transfusion as a source material to isolate functional p-EVs, as already shown experimentally to be potentially feasible for the repair of corneal endothelium cells [220], can contribute to avoiding the wastage of precious PC donations and improving the sustainability of blood establishments.

### Perspectives: challenges and solutions in using platelet-derived biomaterials and biotherapies and current solutions

The fact that allogeneic PCs are a therapeutic cellular product for transfusion presents opportunities for their repositioning towards regenerative medicine and cell therapy. However, several limitations commonly associated with human-derived platelet source materials need to be addressed to propose potential solutions and valorize the potential. One limitation is the relative variability observed in platelet quality, composition, and functional characteristics, which can impact the consistency and efficacy of platelet-derived products. To address this, rigorous exclusion criteria and screening of donors accepted for donations, as employed by blood establishments for transfusion purposes, can ensure a more-homogeneous donor pool. Additionally, standardized processing and quality control protocols can minimize batch-to-batch variations in PCs eventually used to make platelet-derived biomaterials. Pooling an adequate number of platelet donations further contributes to normalizing the characteristics of the starting platelet pool for HPL or p-EV production.

It is well-established that platelets are sensitive to storage conditions, potentially impacting the release of trophic factors and EVs. Although this release might not be detrimental to the production of some HPLs or EVs, careful monitoring of storage conditions is crucial to ensure reproducibility and maintain product specifications. Cryopreservation has shown promise in enhancing the shelf life of PCs for lysate production, but its suitability for p-EV preparation requires validation.

The field of allogeneic platelet-derived biomaterials and biotherapies is still evolving, and standardization of production methods, characterization assays, and regulatory frameworks is needed. Lack of standardized protocols and regulations can pose challenges for product development, clinical translation, and regulatory approval. Collaboration among researchers, clinicians, blood establishments, industry stakeholders, and regulatory bodies is essential to establish standardized protocols, characterization methods, and guidelines. Harmonization of regulatory requirements across jurisdictions can streamline approval processes and leverage the expertise of the blood and plasma product industry.

Ensuring an adequate supply of PCs is crucial for scaling up the production of PLs and p-EVs to meet clinical demands supported by scientific and clinical evidence. Donor recruitment and retention strategies, as well as optimizing the utilization of whole blood units, can help maintain a steady supply. Dedicated platelet collection procedures and the wider introduction of pathogen-reduction technologies can further enhance the availability of PCs, including outdated units that would otherwise be discarded. Cost optimization strategies, such as streamlining manufacturing processes and leveraging economies of scale, are essential for improving accessibility and affordability of platelet-derived products.

Robust research studies, well-designed preclinical and clinical trials, and evidence-based guidelines are crucial for justifying the clinical applications of HPLs and p-EVs. High-quality clinical data supporting their efficacy and safety will guide their use and integration into standard therapeutic approaches. Tailored preparations may be required to address the specificities of different clinical targets.

Addressing the challenges and limitations associated with platelet-derived biomaterials and biotherapies will require collaboration among various stakeholders. By streamlining production processes, sharing resources and expertise, facilitating technology transfer, and ensuring coordinated efforts, the accessibility, affordability, and appropriate clinical use of these therapies can be improved. Collaboration among blood establishments, research institutions, industry stakeholders, and regulatory bodies will play vital roles in overcoming these challenges and maximizing patient access to platelet-derived biotherapies.

## Conclusions

Allogeneic clinical-grade PCs collected from repeat healthy blood donors by licensed blood establishments represent a readily available source of therapeutic cells, which, when not used to cover the needs for hospital transfusion, can serve rapidly growing clinical needs for cell therapy, regenerative medicine, and drug delivery. The beneficial use of allogeneic platelets in these therapeutic domains is currently illustrated by the demonstration that HPL is a safe and potent xeno-free substitute for FBS as a growth medium supplement for the clinical-grade expansion of human cells for transplantation, primarily MSCs, and potentially others like chondrocytes, DCs, or CAR-T cells. Currently, the use of allogeneic PCs as source material for the preparation of HPLs for direct clinical use is still at a relatively early stage; however, recent developments are demonstrating that allogeneic HPLs are a viable and safe alternative to traditional serum eye drops for the treatment of ocular surface disorders, including dry eye syndrome. One can logically anticipate that future preclinical and clinical studies will examine the possibility of using allogeneic HPLs in other degenerative pathologies such as treating recalcitrant skin wounds and degenerative joints, and even as biotherapy to modulate neurodegenerative diseases of the central nervous system. When considering such applications in regenerative medicine, it will remain vital to develop dedicated and standardized HPL preparations, which through careful scientifically based in vitro characterization and well-designed preclinical and clinical evaluations are demonstrated to be safe and best fit the clinical specificities of the pathology addressed for optimal efficacy. In this manuscript, we also highlighted the exciting potential offered by allogeneic PCs and HPLs as source materials, complementary to MSCs, for the isolation of various types of pooled p-EV preparations as subcellular therapeutic modalities for regenerative medicine or as vehicles for drug delivery. Establishing p-EVs as a recognized therapy requires, as for most EV sources [356], the resolution of various manufacturing and regulatory challenges. Challenges include, among others, the development of reliable and dedicated p-EV generation and downstream purification processes, including pathogen-reduction treatments, the capacity to provide suitable biochemical characterization of the molecular cargo and mechanism of action and/or targeting, the development of meaningful quality control assays to guarantee batch-to-batch consistency, safety, and efficacy, and the implementation of meaningful preclinical tests relevant to targeted clinical indications. In conclusion, we believe that allogeneic platelet-derived preparations are likely to have an increasingly prominent position in the future among the arsenal of biotherapies available for cell therapy, regenerative medicine, and drug delivery.

## Data Availability

All materials are available from the corresponding author.

## Abbreviations

ADP: Adenosine diphosphate
ATP: Adenosine triphosphate
BDNF: Brain-derived neurotrophic factor
b-FGF: Basic fibroblast growth factor
CAR: Chimeric antigen receptor
CCL5: C–C motif chemokine ligand 5
CD: Cluster of differentiation
CEC: Corneal endothelium cells
T_CM_: Central memory T-cells
CTGF: Connective tissue growth factor
CXCL4: C-X-C chemokine ligand 4
DC: Dendritic cell
DDS: Drug-delivery system
EDHO: Eye drops of human origin
EGF: Epidermal growth factor
EV: Extracellular vesicle
FBS: Fetal bovine serum
FGF: Fibroblast growth factor
GMP: Good manufacturing practice
HAV: Hepatitis A virus
HBV: Hepatitis B virus
HCV: Hepatitis C virus
HGF: Hepatocyte growth factor
HIV: Human immunodeficiency virus
HPL: Human platelet lysate
HUVEC: Human umbilical vein endothelial cell
IGF-I and II: Insulin-like growth factor I and II
IL: Interleukin
LAMP: Lysosomal-associated membrane protein
MI: Myocardial infarction
MSC: Mesenchymal stromal cell
NETs: Neutrophil extracellular traps
OSC: Open canalicular system
PAS: Platelet additive solution
PC: Platelet concentrate
PDGF: Platelet-derived growth factor
PF4: Platelet factor 4
PL: Platelet lysate
PRP: Platelet-rich plasma
PSGL: P-selectin glycoprotein ligand
RBC: Red blood cell
S/D: Solvent/detergent
SED: Serum eye drop
*SOX9*: SRY-box transcription factor 9
TDDS: Targeted drug delivery system
TNF: Tumor necrosis factor
TGF: Transforming growth factor
UV: Ultraviolet
VEGF: Vascular endothelial growth factor
vWF: Von Willebrand factor
WBC: White blood cell
